# Cerebellar connectivity maps embody individual adaptive behavior in mice

**DOI:** 10.1038/s41467-022-27984-8

**Published:** 2022-01-31

**Authors:** Ludovic Spaeth, Jyotika Bahuguna, Theo Gagneux, Kevin Dorgans, Izumi Sugihara, Bernard Poulain, Demian Battaglia, Philippe Isope

**Affiliations:** 1 Institut des Neurosciences Cellulaires et Intégratives, CNRS, Université de Strasbourg, 67084 Strasbourg, France; 2 Aix-Marseille Université, Institut de Neurosciences des Systèmes, CNRS, 13005 Marseille, France; 3 Department of Systems Neurophysiology, Tokyo Medical and Dental University Graduate School of Medical and Dental Sciences, 1-5-45 Yushima Bunkyo-ku, Tokyo, 113-8519 Japan; 4 University of Strasbourg Institute for Advanced Studies (USIAS), 67084 Strasbourg, France; 5 Present Address: Dominick P Purpura Department of Neuroscience, Albert Einstein College of Medicine, Bronx, NY USA; 6 Present Address: Department of Psychology, Carnegie Mellon University, Pittsburgh, PA USA; 7 Present Address: Okinawa Institute of Science and Technology, Graduate University of Okinawa, Onna, Japan

**Keywords:** Cellular neuroscience, Neural encoding, Cerebellum

## Abstract

The cerebellar cortex encodes sensorimotor adaptation during skilled locomotor behaviors, however the precise relationship between synaptic connectivity and behavior is unclear. We studied synaptic connectivity between granule cells (GCs) and Purkinje cells (PCs) in murine acute cerebellar slices using photostimulation of caged glutamate combined with patch-clamp in developing or after mice adapted to different locomotor contexts. By translating individual maps into graph network entities, we found that synaptic maps in juvenile animals undergo critical period characterized by dissolution of their structure followed by the re-establishment of a patchy functional organization in adults. Although, in adapted mice, subdivisions in anatomical microzones do not fully account for the observed spatial map organization in relation to behavior, we can discriminate locomotor contexts with high accuracy. We also demonstrate that the variability observed in connectivity maps directly accounts for motor behavior traits at the individual level. Our findings suggest that, beyond general motor contexts, GC-PC networks also encode internal models underlying individual-specific motor adaptation.

## Abstract (web summary)

The variability in synaptic connectivity observed at the cerebellar granule cell - Purkinje cell connection in mice accounts for motor behavior traits at the individual level, suggesting that cerebellar networks encode internal models underlying individual-specific motor adaptation.

## Introduction

Sensorimotor adaptation and motor learning rely on a combination of neuronal computations performed in different brain areas which communicate through cerebello-thalamo-cortical loops^1–7^. Population dynamics in cortical networks encoding stimulus features route information to different parts of the brain dedicated to motor planning and motor control. If these neuronal dynamics eventually succeed in producing an adapted behavior, they are stabilized through synaptic plasticity yielding to a mutual structuring of circuit connectivity and activity patterns across the brain^8–11^. Recent experiments described how different types of neurons in the cerebral cortex are selectively and functionally connected depending on stimulus features they have to encode^12–15^. Similarly, in the cerebellar cortex, structured synaptic maps across individuals have been described in identified modules^16,17^. However, how they are related to adaptive behavioral conditions is not well understood.

One of the major roles of the cerebellum in sensorimotor adaptation is to learn to predict the sensory feedback of motor commands using stored and adaptable internal models of body coordinates^18–21^. By computing the actual sensory feedback, cerebellar networks estimate prediction errors, which are then used to adapt the ongoing motor sequence and internal models. Indeed, many studies demonstrated that the cerebellum can learn and store complex adaptive behavior^22,23^, such as walking on a split-belt treadmill^24,25^. However, how locomotor adaptation is encoded in cerebellar cortical synapses is unknown. One hypothesis would be that synaptic connectivity maps encode movement features in each specific context (e.g., limb movement when walking or running). Connectivity maps would then represent individual-specific engrams of adaptive behaviors established throughout motor learning and such feature-based maps would be animal and context specific^20,21^. To address this hypothesis, we investigated whether functional synaptic maps between granule cells (GC) and Purkinje cells (PC) in the anterior cerebellar vermis, an area involved in adaptation of locomotion^26–30^, are modified in different locomotor contexts (after training in a wheel or following impairment of locomotion) and during development. Many studies suggest that GC-PC synapses are a major site of sensorimotor information storage in the cerebellar cortex^17,22,31,32^. GCs integrate sensorimotor information conveyed by mossy fibers (MFs) and carry motor commands or current body sensory state from many precerebellar nuclei^33–36^. GCs compute and distribute this information to many different PCs via their long axons, the parallel fibers (PFs), leading to specific excitatory, functional connectivity maps^16,17^. The second major excitatory input to the cerebellar cortex, the climbing fibers (CF), target PCs, controls plasticity at the GC-PC synapses and its topographical organization defines an array of anatomo-functional modules called microzones^37–43^ (Supplementary Fig. 1).

We used a combination of electrophysiological recordings and glutamate uncaging to establish GC-PC functional connectivity through excitatory synaptic maps and described their spatial organization using a graph-based description of synaptic weights. Through functional reconstruction, graph representations, and the quantification of their graph-theoretical features, we showed that the behavioral context leaves a trace in the spatial organization of maps. We also found that while connectivity maps are correlated to behavioral conditions, each mouse developed a specific individual combination of connectivity traits linked to its individual and highly specific locomotor activity, suggesting that behavior may causally shape the spatial (re)organization of functional connectivity maps, biased but not fully determined by somatotopic hardwiring.

## Results

### GC-PC functional synaptic connectivity maps in the cerebellar cortex

**Fig. 1:**
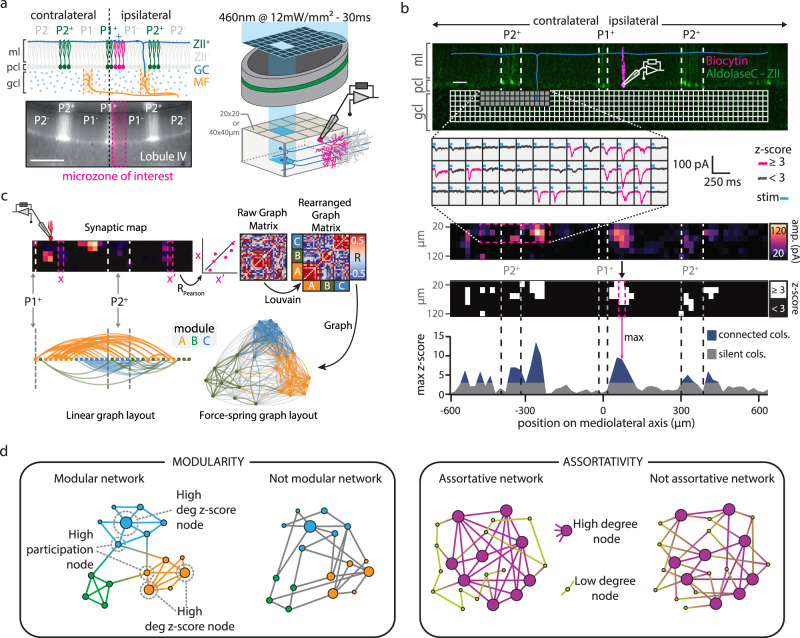
Description of GC-PC synaptic maps and profiles. **a** Schematic diagram (top left panel) and corresponding picture of an acute slice from an ALDOC-Venus mouse (bottom left panel) illustrating cerebellar cortical anatomy. Recorded PCs (red) are close to the midline (<130 μm) and belong to the P1^−^ microzone of lobule III/V. Zebrin II positive (ZII^+^, green) and ZebrinII negative (ZII^−^, light gray) PCs coincide with microzonal boundaries (Supplementary Fig. 1). MFs (orange) project onto columns of GCs. Scale bar = 300 μm. Right panel, photo-stimulation apparatus for glutamate uncaging using patterned blue light illumination (site size: 20 × 20 or 40 × 40 μm). PCs were whole-cell patch-clamped. gcl, granule cell layer; ml, molecular layer; pcl, Purkinje cell layer. **b** Building GC-PC synaptic maps and profiles. Upper part, post-hoc reconstruction of an acute slice. green, endogenous aldolase-C-Venus fluorescence; magenta, recorded PC; scale bar = 50 μm. Light-evoked EPSCs from each stimulation site were averaged. Significant EPSCs are shown in magenta (*z*-score ≥ 3). Lower part, resulting synaptic maps are translated into *z*-score maps (Methods and Supplementary Fig. 2). Maximal z-score value in each GCL column (magenta-dashed box) along the mediolateral axis was projected on a synaptic profile. Blue areas show GC columns connected to the recorded PC. **c** Connectivity map preprocessing for graph-network analyses. Column-wise Pearson correlations (e.g., x and x′ columns) of synaptic weights lead to a spatial correlation matrix (Raw graph matrix). The raw matrix was rearranged using the Louvain community detection algorithm giving the Rearranged graph matrix with well-defined graph modules (A, B, C). The resulting graph can be visualized using a force-spring algorithm (Force spring layout,) or according to node positions along the mediolateral axis, thus respecting the original spatial arrangement rather than using an arbitrary space (linear layout; Methods section). **d** Schematic description of graph parameters (see Supplementary Fig. 4). Networks can be characterized by partitioning them into modules and assessing modularity. Detailed information on modular organization is obtained evaluating module degree *z*-score, (highlighting strong hubs organizing connectivity within modules) or participation (highlighting nodes serving as interfaces between modules). Assortativity, illustrates whether strongly (weakly) connected nodes tend to connect with other strongly (weakly) connected nodes. These metrics capture different specific traits of the map spatial organization.

We established functional synaptic connectivity maps between GCs and PCs in acute cerebellar slices of the anterior vermis (lobules III to V) and selected a group of medial PCs (0–130 µm from midline^17^, Fig. 1a and Supplementary Fig. 1). To enable PC recordings at the same location in different slices and mice, we took advantage of the specific expression of a family of neurochemical markers (e.g .zebrin II)^39^ in subsets of PCs arranged in parasagittal stripes. These markers, which are highly conserved between individuals, delimit zebrin positive (e.g. P1^+^ and P2^+^) and negative (e.g., P1^−^ and P2^−^) bands of PCs matching with the topographical arrangement of the CF inputs^43,44^, outlining cerebellar microzones in the cortex (Supplementary Fig. 1). PCs were then whole-cell patch-clamped in P1^−^ bands of AldolaseC-Venus fluorescent transgenic mice^45^ (*N* = 84 mice, Table 1) expressing Venus in PCs of zebrin II positive bands (Fig. 1a). Small groups of GCs were sequentially activated (in grids of 128 or 384 square sites of 40 × 40 µm or 20 × 20 µm, yielding low- and high-resolution photostimulation respectively) using Rubi-Glutamate uncaging^46^ while synaptic excitatory currents (EPSCs) were recorded in PCs (*n* = 153 PCs, Fig. 1a, b, Supplementary Fig. 2a, b, and Table 1; Methods section). For each PC, a unique GC-PC functional map was built from the averaged EPSCs elicited by each of the 128 or 384 photostimulated sites (Fig. 1b; Methods section). To define whether a site is functional or silent, EPSC amplitudes were normalized to noise level and expressed as z-scores^17^ (i.e., *z*-score ≥3 was considered an active site; Fig. 1b and Supplementary Fig. 2; Methods section). MF inputs originating in a specific precerebellar nuclei project onto GCs in most of the GC layer height and at several locations in a lobule, defining a fractured and patchy columnar somatotopy (Supplementary Fig. 1; see refs. ^34,47–49^). We therefore treated the GC layer either as a grid or as a series of GC columns of 40 or 20 µm width corresponding to either low or high-resolution photostimulation, respectively. Each functional connectivity map was then represented either as a map or a projected synaptic profile of the maximal synaptic weight in each GC column and aligned to the zebrin bands (Fig. 1b and Supplementary Fig. 3; and Methods section).

**Table 1:** Group description and experimental conditions.

Condition	*N*_mice_	*n*_maps_	Description	Mapping resolution (µm)
Pups (PND9–10)	5	11	Mice raised in standard conditions. Maps were recorded between PND9-10	40 × 40
Juvenile (PND12–13)	6	12	Mice raised in standard conditions. Maps were recorded between PND12-13	
Adolescent (PND14–18)	7	15	Mice raised in standard conditions. Maps were recorded between PND14-18	
Adults (PND > 30)	7	10	Mice raised in standard conditions. Maps were recorded after PND-30	
Control	9	14	Mice raised in standard conditions. Maps were recorded after PND-30	20 × 20
Short training	7	13	7 daily & consecutive sessions in the running wheel. Maps were recorded 7–8 days after training start	
Long training	6	11	19 daily & consecutive sessions in the running wheel. Maps were recorded 19–20 days after training start	
Early sham	7	11	Cuff surgery, no cuff. Maps were recorded 2–9 days after surgery	
Early cuff	13	25	Cuff surgery + cuff. Maps were recorded 2–9 days after surgery	
Adapted sham	9	14	Cuff surgery, no cuff. Maps were recorded 28–35 days after surgery	
Adapted cuff	8	17	Cuff surgery + cuff. Maps were recorded 28–35 days after surgery	
Total	84	153	–	–

We also developed an analytical method to investigate how GC columns connected to a given PC relate to each other, accounting for the fractured and patchy somatotopy of MF projections. To do this, we used an analytic workflow based on a mathematical graph representation of the functional maps (Fig. 1c, d, Methods section). Graphs (or networks) are generic abstract entities composed of nodes and links between them^50^. We considered each map as a matrix *M*, whose *M*_*xy*_ is the mean EPSC amplitude recorded at the GC site coordinates (*x,y*) and *C*_*x*_ the column at coordinate *x* of a stimulation grid (Fig. 1c; one column = one node of the graph network). We computed the normalized Pearson correlations (*C*_*xx*’_) for every pair of columns *x* and *x*′ along the mediolateral axis of the functional connectivity maps. *C*_*xx*__′_ entries become the weights of edges between graph nodes associated to the positions *x* and *x*′. *C*_*xx*__′_ values were compiled in a correlation matrix C(M) (Raw Graph Matrix in Fig. 1c) which can be considered as the adjacency matrix of an undirected weighted graph (Methods section). GC columns belonging to one or multiple patches will have similar synaptic profiles, and the corresponding graph nodes will thus be strongly connected, forming network modules. These modules are highlighted as matrix blocks, appearing in the adjacency matrix after sorting its rows and columns according to a Louvain community detection algorithm^51,52^ (Re-arranged Graph Matrix in Fig. 1c). In Fig. 1c, nodes belonging to three different modules have been colored into two alternative visualizations of the graph associated with a single map. A linear layout (Fig. 1c, left), in which node positions follow the original mediolateral axis alignment illustrates that modules tend to be composed of spatially contiguous nodes, with some exceptions. Therefore, spatially distant GC columns can belong to the same graph module, accounting for MF fractured somatotopy and the multimodal MF inputs on individual GC columns^35,36^. An alternative network layout was optimized for module visualization by a force-spring algorithm (Fig. 1c, right). In this representation, modules of strongly connected nodes appear as clusters of tightly interconnected nodes at close spatial distance.

**Fig. 2:**
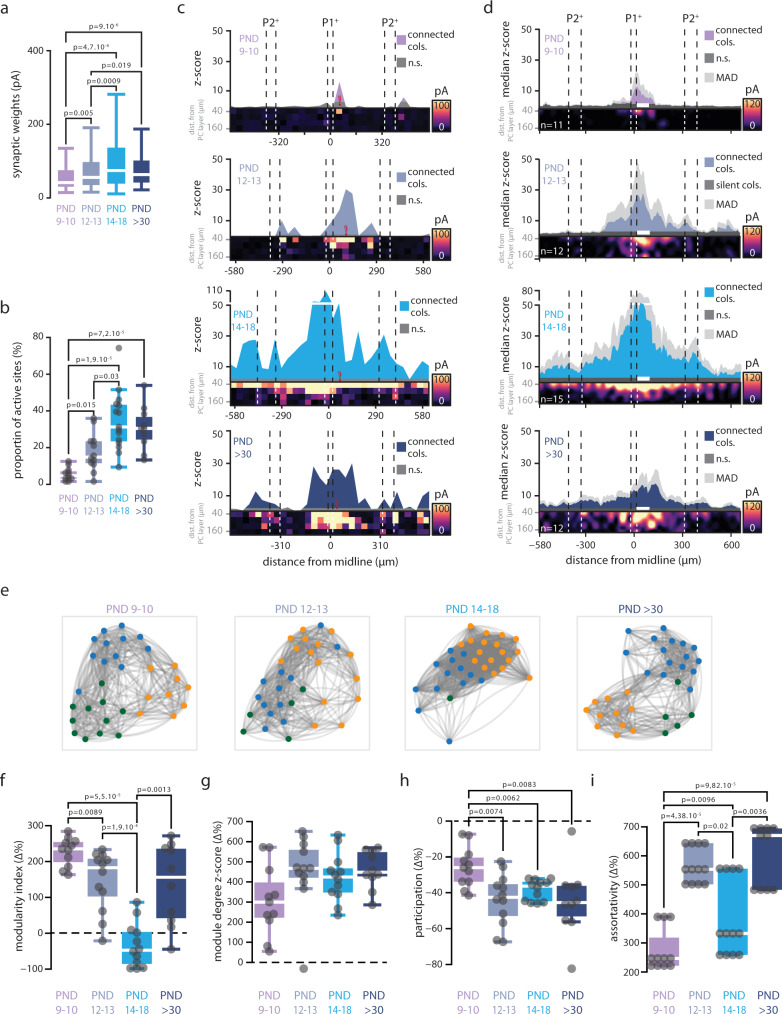
Postnatal development of GC-PC synaptic maps. **a** Distribution of GC-PC significant synaptic weights (z-score ≥ 3) during postnatal development. Mean ± SD in pA: PND9-10, 62 ± 55, *n* (GC sites/maps/mice) = 78/11/5; PND12-13, 87 ± 94, *n* = 271/12/6; PND14-18, 113 ± 128, *n* = 647/15/7; PND > 30, 92 ± 88, *n* = 392/10/7. Whisker bounds: minima/maxima, median, interquartile range. Kruskal-Wallis test (KW, all conditions), *p* = 1.99.10^−11^. Mann–Whitney *U* (MWU) post-hoc, *p*-values in graph. *P*-value > 0.05 in Source Data. **b** Proportion of GC active sites (*z*-score ≥ 3) measured in GC-PC maps. Mean ± SD per slice in %: PND9-10, 5.5 ± 3.5, *n* (maps) = 11; PND12-13, 17.6 ± 10, *n* = 12; PND14-18, 34.7 ± 15.6, *n* = 15; PND > 30 30.6 ± 11.4, *n* = 10. KW (all conditions), *p* = 4 × 10^−6^. MWU, *p*-values in graph. *P* > 0.05 in data source. Whisker bounds: minima/maxima, median, interquartile range. **c** Examples of synaptic profile (top of each subpanel) and synaptic maps (bottom of each subpanel) at PND9-10, PND12-13, PND14-18, and in adult mice. The red PC indicates the position of the recorded PC. Connected cols: *z*-score ≥ 3 in at least one site of the column; n.s.: *z*-score < 3 in all sites of the columns. **d** Median synaptic profiles (top of each subpanel) and averaged synaptic maps (bottom of each subpanel) recorded at PND9-10, PND12-13, PND14-18, and in adult mice. The white bar represents the averaged position of the recorded PCs. Median absolute deviation (MAD) is shown in light gray. **e** Force-spring graph representation of example maps at PND9-10, PND12-13, PND14-18, and in adult mice. Nodes of a given color belong to the same module. **f**, **g**, **h**, **i** Graph properties (modularity index, module degree *z*-score, participation, and assortativity respectively) of GC-PC synaptic maps, expressed relative to the median of chance-level distributions (∆%; see Methods section). Values above (below) the dashed horizontal line at 0 represent an over- (under) representation of the considered connectivity trait compared to its null-model counterpart. Whisker bounds: minima/maxima, center: median, box: interquartile range. Modularity (mean ± SD in Δ%): PND9-10, 228 ± 40, *n*(maps) = 11; PND12-13, 146 ± 58, *n* = 12; PND14-18, −34 ± 61, *n* = 15; PND > 30, 137 ± 112, *n* = 10. KW (all conditions), *p* = 1.03 × 10^−5^. MWU, *p*-values in graph. Module degree z-score (mean ± SD in Δ%): PND9-10, 308 ± 172; PND12-13, 453 ± 177; PND14-18, 422 ± 120; PND > 30, 460 ± 94. KW (all conditions), *p* = 0.076. Participation (mean ± SD in Δ%): PND9-10, −26 ± 11; PND12-13, −44 ± 15; PND14-18, −39 ± 6; PND > 30, −45 ± 19. KW (all conditions), *p* = 0.0053. MWU, *p*-values in graph. Assortativity (mean ± SD in Δ%): PND9-10, 277 ± 73; PND12-13, 566 ± 61; PND14-18, 385 ± 132; PND > 30, 605 ± 105. KW (all conditions), *p* = 7.68 × 10^−6^. MWU, MWU, *p*-values in graph. *P* > 0.05 in Source Data. Source data are provided as a Source Data file.

**Fig. 3:**
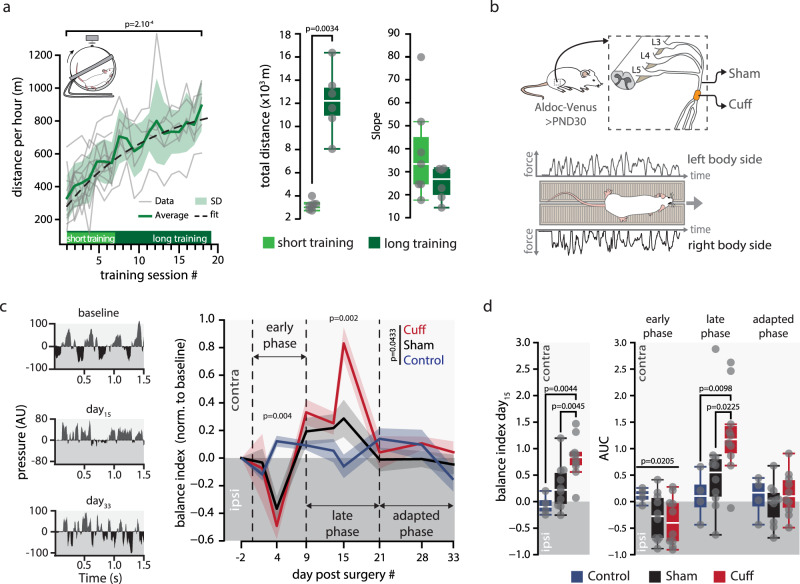
Locomotor adaptation in different contexts. **a** Left panel, distance traveled by mice in the wheel across sessions. Short training: 1 h/day for 7 consecutive days, *n*(mice) = 7; long training: 1 h/day for 19 consecutive days, *n* = 6. Two-tailed paired *t*-test between training session #1 and training session #19, *p*-value in graph. Dashed line, logistic regression, *p* = 2.75 × 10^−26^. Right panels, total distance and slope of the distance across sessions for short (*n* = 6) and long (*n* = 7) training animals. Total distance mean ± SD in km: short training, 3.1 ± 0.4; long training, 12.2 ± 2.6. Slope mean ± SD in m/session: short training, 38.6 ± 19.8; long training, 24.9 ± 6.7. Whisker bounds: minima/maxima, median, interquartile range. Two-sided MWU, *p*-value in graph. *P* > 0.05 in Source Data. **b** Upper panel, cuff model of locomotor impairment. In cuff animals, a 2-mm-long polyethylene cylinder (cuff) was surgically wrapped around the main branch of the right sciatic nerve. Sham animals underwent only surgeries. Lower panel, illustration of a trial on the force pressure corridor. **c** Balance deficits in cuff (*n* = 10) and sham (*n* = 10) groups. Left panel, examples of raw data measurements from the force-sensor corridor in a cuffed mouse before surgery (baseline), 15 days after surgery and 33 days after surgery. AU, arbitrary unit. Balance Index (BI) corresponds to the log of the ratio of the integrated force-signal from each side of the body. Right panel, averaged (±SEM) time-course of the BI in cuffed (*n* = 10, red), sham (*n* = 10, black), and controls (*n* = 4, blue). Values were normalized to baseline. Early phase, from day 0 to day 9 post surgery; late phase, from day 9–21 post surgery; adapted phase, from 21 days post-surgery. One-way repeated measurements MANOVA from day 2–28, *F* =2.16; post hoc ANOVA, early phase/late phase, *F* = 7.17/*F* =8.23, *p*-values in graph. **d** Left panel, normalized BI measured at Day 15 post surgery. Mean ± SD: control, −0.06 ± 0.19 (*n* = 4); cuff, 0.83 ± 0.37 (*n* = 10); sham, 0.29 ± 0.43 (*n* = 10). Whisker bounds, same as in a. KW (all groups), *p* = 0.00354; post hoc two-sided MWU, *p*-values in graph. Right panel, area under the curves (AUC) of balance time course shown in c for cuffed, sham and control groups. Mean ± SD in early phase: control, 0.1 ± 0.1; cuff, −0.4 ± 0.4; sham, −0.3 ± 0.4. Mean ± SD in late phase: control, 0.1 ± 0.4; cuff, 1.3 ± 0.8; sham, 0.6 ± 0.9. Mean ± SD in adapted phase: control, 0.08 ± 0.3; cuff, 0.14 ± 0.4; sham, −0.03 ± 0.4. Whisker bounds same as in **a**. Levene’s test, early phase, *p* = 0.0205; two-sided MWU, *p*-values in graph. *P* > 0.05 in Source Data. Source data are provided as a Source Data file.

**Fig. 4:**
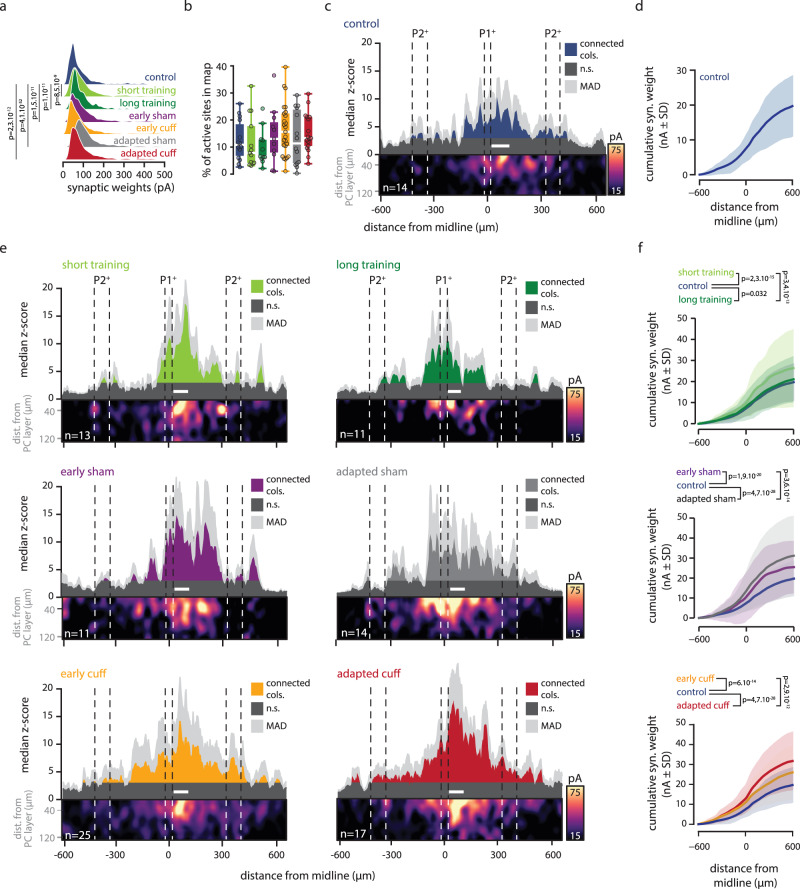
Synaptic GC-PC maps and profiles during and after locomotor adaptation. **a** Distributions of GC-PC significant synaptic weights recorded in each behavioral condition. Whisker bounds: minima/maxima, median, interquartile range. Mean ± SD in pA; control, 72.3 ± 50, *n* (sites/maps/mice) = 588/14/9; short training, 89.8 ± 71, *n* = 481/13/7; long training, 86.7 ± 48.3, *n* = 375/11/6; early sham, 91.9 ± 60, *n* = 534/11/7; early cuff, 78.7 ± 62, *n* = 1582/25/13; adapted sham, 116.2 ± 69, *n* = 581/14/9; adapted cuff, 88.8 ± 59, *n* = 985/17/8. KW (all conditions), *p* = 1.27 × 10^−71^. Two-sided MWU with control group, *p*-values in graph. *P*-values > 0.05 in Source Data. **b** Proportion of active sites in maps from each condition. Whisker bounds as in previous figures. Mean ± SD in %: control, 12.4 ± 7, *n* = 14; short training, 11.6 ± 9, *n* = 13; long training, 9.9 ± 6, *n* = 11; early sham, 14.2 ± 10, *n* = 11; early cuff, 16.5 ± 9, *n* = 25; adapted sham, 13.3 ± 9.8, *n* = 14; adapted cuff, 15.3 ± 7, *n* = 17. KW test (all conditions), *p* =  0.55. **c** GC-PC synaptic connectivity maps recorded in control mice. Upper part of the panel, median synaptic profile. White bar, averaged position of recorded PCs. In dark blue, connected columns (connected cols); in dark gray, non-connected columns (n.s.); in light gray, median absolute deviation (MAD). Lower part of the panel, averaged synaptic map. **d** Cumulative strength (from synaptic profile) of evoked response along the mediolateral axis in the control group. Mean ± SD in nA at 600 µm: 19.7 ± 8.57 (*n* = 14). **e** Same as in **c** for short & long training, early/late sham, and early/late cuff groups. White bar, averaged position of recorded PCs. In dark gray, non-connected columns (n.s.); in dark colors, connected columns (connected cols); in light gray, MAD. **f** Cumulative strength (from synaptic profiles) of evoked response along the mediolateral axis in each condition and compared to the control group (in blue). Mean ± SD in nA at 600 µm (same n as above): short training, 26 ± 18.6; long training, 21.2 ± 11; early sham, 25.5 ± 13.2; early cuff, 26 ± 13.5; adapted sham, 31.2 ± 20; adapted cuff, 31.8 ± 14. Two-tailed KS test, *p*-values in graph. *P* values were corrected with Holm method for multiple comparison. Source data are provided as a Source Data file.

We then quantified the topology of these graph representations of the synaptic maps using 4 standard metrics (Fig. 1d, Supplementary Fig. 4, and its supplementary note; Methods section). (1) The modularity index is a measure of how modular a graph is, eventually related to the “patchiness” of the associated GC-PC synaptic map. (2) The average module degree *z*-score quantifies whether the connectivity of a node to its neighbors within the same module is stronger (or weaker) than average. This metric can detect degree heterogeneity within modules, possibly reflecting the existence of patches in the associated map with simpler or more complex shapes. (3) The average participation coefficient measures the probability that a node in a module is also connected to nodes in other modules. This metric may capture the tendency of patches in the map to partially overlap. (4) The average local assortativity measures the tendency of nodes to connect to other nodes with similar strength (i.e., the sum of weights of connections to a node). Strong assortativity in a map may correspond to the co-existence of narrower and broader patches. Together these abstract metrics convey concrete and complementary information about the geometry of the connectivity maps. A detailed illustration of how synaptic map traits are translated into graph-level features is given by Supplementary Fig. 4, which also provides examples of maps with relatively larger or smaller graph feature values. In the following, unless otherwise specified, we will show graph metrics values as normalized percent differences (denoted by the ∆% symbol) with respect to chance level expectations (see Methods). Significantly positive (or negative) ∆% values indicate non-trivial over- (or under)-expression of a given graph property as compared to its null counterpart. Such relative normalization allows easier comparison of maps sampled at different resolutions. Using functional activation and graph-based properties, we assessed how functional connectivity maps change during postnatal development (Fig. 2) and whether they are modified after an injury (right-hindlimb impairment via sciatic nerve cuffing) or locomotor training in a running wheel (Figs. 3 and 4, Table 1, Methods section).

### Network connectivity rules mature during development

We first addressed how GC-PC functional connectivity maps were established and structured during normal postnatal development. If these maps reflect the establishment of the input/output relationships in a topographic manner, we expect to observe a progressive and regular evolution of their structuration as GCs migrate from the external granule cell layer during the first three weeks after birth and connect to MFs^53^. However, key periods of locomotor adaptation have been observed in postnatal development of mice. For example, mice open their eyes and start walking between postnatal day 12 (PND12) and PND18^54–56^. At this age, they are hyperactive with bouts of vigorous jumping called *‘*hoppy’ or *‘*popcorn’ stage resulting from synchronous contraction of fore and hind limb extensors^55,57^. To assess whether these behavioral features correlate with specific synaptic map adaptation, we recorded GC-PC functional connectivity maps at low resolution (40 × 40 µm site area, 128 sites per grid) at different time points during mouse development: from PND9-10, before proper quadrupedal locomotor activity, to young adults (PND > 30) when locomotion is well adapted (Fig. 2 and Table 1). We observed that EPSC amplitudes elicited by individual GC sites increased between PND10 and PND14-18, while no significant differences were observed between PND14-18 and adulthood (Fig. 2a). In addition, the number of active sites increased linearly by 7-fold between PND9-10 and PND14-18, then remained stable in adults (Fig. 2b). This increase in functional synaptic connectivity may be the result of new functional GC-PC synapses rather than a change in GC excitability as no difference could be observed in GC firing rates between pups and PND14 following glutamate uncaging (Supplementary Fig. 5a).

However, the evolution of the spatial and structural organization of the maps follows different rules. At PND9-10, functional GC sites were mostly observed below the recorded PC (Fig. 2c, d), nonetheless distant GC inputs were already, albeit rarely, observed (Fig. 2c) as PF lengths in the medial vermis already exceed several hundred µm long (Supplementary Fig. 5b). A patchy organization (i.e., patches of functional sites surrounded by silent ones) was observed at PND12-13 and after PND30 (Fig. 2c, d). By aligning synaptic maps on zebrin bands (Supplementary Fig. 3 and Methods section), the median synaptic profile was computed for each group of mice (Fig. 2d) and showed that the distributions of functionally connected GC columns were not randomly distributed with multiple hotspots of connectivity or silent areas at specific locations as already observed in a previous study^17^. On the contrary, at PND14-18, both synaptic maps and profiles were homogeneous, and almost all GC columns appeared functionally connected to the recorded PC (Fig. 2d).

To quantify the degree of patchiness and the structure of the maps, we calculated the four relative graph metrics (Fig. 1d and Supplementary Fig. 4, Methods section) for each synaptic map during development (Fig. 2e–i). Overall, GC columns are organized in distinct modules with a high modularity index and module degree *z*-score in average as these two graph metrics are well above chance level values (positive ∆% values, Fig. 2f, g). Confirming the existence of well-separated patches in the map, participation coefficients were significantly smaller than the chance level at all developmental stages (negative ∆% values, Fig. 2h). Assortativity, on the contrary, was always strikingly above chance level (Fig. 2i), like modularity and degree *z*-score, illustrating patch diversity and specificity.

Nevertheless, graph metrics varied with age. Modularity index (Fig. 2f) first decreases, then recovers to near original values in adults. The participation coefficient at all ages was significantly lower than the values for PND9-10 (Fig. 2h), and the local assortativity showed a non-monotonic change with age (Fig. 2i). Altogether, these changes suggest that graph modules in adults became more compact, better defined, and overlapped less (see Supplementary Fig. 4). On the contrary, during the PND 14-18 epoch, modularity and assortativity indexes reached transitory minimal value (Fig. 2f, i). These results indicate that, at this age, boundaries between patches are blurry with responses organized around a center with a maximum amplitude as if only one extended module persisted (i.e. the ∆% modularity index is close to chance level, Fig. 2f). These findings suggest a loss of network structure at this critical age. Therefore, as suggested by functional connectivity maps (Fig. 2d), analysis of graph properties showed that network properties do not mature linearly with age. Rather, they display denser and relatively unstructured functional connectivity in PND14-18 mice, resuming the appearance of a more structured patchy functional connectivity at adulthood. Altogether, these results suggest that connectivity maps are not built solely by following strict anatomical input/output rules, but likely rely on adaptive mechanisms occurring during development.

### Adapted locomotor activity correlates with specific connectivity maps

Development leads to adult functional connectivity maps sharing spatial similarities, which may illustrate that, in normal conditions, locomotion is a conserved behavior. We now asked whether different locomotor contexts (six different contexts and a control group, Table 1) in which adult mice must develop new adaptive behaviors could also re-organize functional synaptic maps. Since locomotor contexts may have subtler effects on maps organization than development, we recorded functional maps at a higher spatial resolution (20 × 20 µm photostimulation sites) and at two different time points during adaptation. (1) Two groups of mice were trained to run in a wheel (1 h/day) for 7 (short training) or 19 (long training) consecutive days (Fig. 3a), learning “gallop”, a new type of gait for animals living in a cage^58^. In the last session, long training mice have traveled 2.8-fold more distance per session compared to first session (Fig. 3a). Synaptic maps were recorded on days 7–8 (short training) or days 19–20 (long training). (2) In two other groups, locomotion was impaired by inserting a cuff around the sciatic nerve of the right hindlimb^59^ (Fig. 3b) and functional connectivity maps were recorded at 2–9 days (early cuff) or after 21 days (adapted cuff) after the surgery, allowing the animals to fully re-adapt their locomotion. Finally, two sham groups underwent only surgeries (i.e., no cuff was inserted) and were recorded at the same time as cuffed mice (early sham and adapted sham). It should however be noted that the sham surgery itself, as it requires manipulation of the sciatic nerve, is likely to induce a mild inflammation in the right hindlimb.

We evaluated locomotor deficits in cuffed and sham mice in a corridor equipped with two streams of force sensors to monitor weights from either side of the body when walking (Fig. 3b, c, Methods section). We defined a balance index (BI) as the log of the ratio of the total weight generated by the left vs the right side of the body along the corridor (Fig. 3b, c). The BI was measured twice a week during one month after cuff or sham surgery. As expected, before surgery, BI index was close to 0 in all mice. After surgery balance was altered in cuffed and sham animals (Fig. 3c). During the first week post-surgery, sham and cuffed mice limp on the right side, which coincide with a negative BI illustrating a higher weight on the side of the surgery (early phase in Fig. 3c). The following week, sham and cuff mice did not recover full balance. BI switched to a positive value, illustrating compensatory behavioral strategies, with a peak two weeks after surgeries (late phase in Fig. 3c). Ultimately, after one month, all mice recovered balance and no apparent locomotor impairment remained (adapted phase in Fig. 3c). While we observed that sham animals quickly recovered their ability to walk after the surgery, balance recovery followed a similar time-course than cuffed animals albeit with a smaller impairment (Fig. 3d). Functional GC-PC maps were then recorded in acute slices either during locomotor re-adaptation (short training, early sham and cuff) or after full adaptation (long training, adapted sham and cuff, Fig. 4).

We first compared maps at the level of inter-group differences (Fig. 4a). We observed that synaptic weights elicited by connected GC sites (*z*-score ≥ 3) were significantly larger in all but early cuff groups when compared to control group (Fig. 4a). However, the overall proportion of active sites per map was not significantly different between conditions (Fig. 4b). We then analyzed the spatial organization of GC-PC synaptic maps. As in the control condition (Fig. 4c), synaptic maps from every group showed a clear patchy organization (Fig. 4e). Subtracting averaged maps from the control condition suggested that, in all groups, most GC sites elicited larger synaptic weights albeit some displayed a decrease (Supplementary Fig. 6a). Median synaptic profiles showed that local GC columns systematically elicited significant inputs and distal hotspots of functionally connected columns were interleaved with non-connected areas as confirmed by a bootstrap analysis (Fig. 4c, e and Supplementary Fig. 3c; Methods section). Even if these profiles presented a substantial degree of inter-individual variability (see Median Absolute Deviation, MAD, dispersions in Fig. 4c, e), locomotor adaptation led to a consistent increase in the group-level cumulative synaptic weights when compared with the control group (Fig. 4d, f). Finally, as seen in cumulative plots of median synaptic profiles from injured mice (sham and cuff), complete behavioral re-adaptation (i.e., adapted sham and adapted cuff groups) was associated with an additional increase in synaptic weights when compared to early conditions (Fig. 4f). Moreover, in adapted cuff animals, in the ipsilateral side of the map, synaptic weights and proportion of active sites were also significantly higher than in the contralateral side (Supplementary Fig. 6b). These results suggest that successful behavioral adaptation following invasive modifications of the locomotor apparatus recruit more GCs or potentiate existing GC-PC synapses.

Altogether, these results demonstrate that behavioral adaptation within different locomotor contexts directly influenced functional connectivity in the cerebellar cortex, inducing noticeable differences already at the level of group median maps, despite large inter-individual differences (Fig. 4e). Such findings may be related to the topographic organization of MF inputs received by GC columns (Supplementary Fig. 1), so that the spatial structure of inputs driving plasticity at the GC-PC synapses is different for alternative contexts, each associated to a specific locomotor adaptation. To test this hypothesis, we addressed whether the anatomical organization of the cerebellar cortex, which reflects the hardwired organization of external inputs, could account for the observed spatial organization of functional connectivity maps. We therefore compared the segmentation of the map in terms of anatomical microzones (structural zones defined by zebrin bands; see Supplementary Fig. 1) with an alternative segmentation derived for each map in terms of its graph representation (functional zones). Notably, we assigned any two locations along the mediolateral axis of the map to the same functional zone if both the following conditions were met: (i) their associated graph nodes belong to the same graph module; and, (ii) all locations between them are also assigned to the same functional zone. Such constructive procedure (see Methods section) guarantees that each map is completely partitioned into contiguous spatial ranges, each of them being a different functional zone (Supplementary Fig. 7). To assess how structural zones accounted for functional zones (potentially completely independent from them), we then quantified the relative mutual information (MI) between the two alternative map-tailored partitions (see Methods section). As shown by Supplementary Fig. 7a, the measured partition overlaps were in the range of 50–57% which is well above chance level for all groups (at most ~39%, 95%, confidence interval, CI, permutation testing; see also Supplementary Fig. 7b for an alternative, more relaxed definition of functional zones confirming the same result). Although significant, this overlap between structural (microzones) and functional zones (graph modules) was far from complete which may illustrate the multimodal origin of MF inputs in each GC layer area. We conclude that maps could be re-shaped by activity-dependent plasticity and are still able to partially override anatomical constraints.

### Graph descriptions of connectivity maps can discriminate different locomotor adaptation conditions

The large inter-individual variability and the non-perfect alignment between functional and structural zones (microzones) may limit the condition-specificity of GC-PC connectivity maps. To verify whether locomotor contexts could still be discriminated even at the level of individual maps, beyond group-level comparisons, we resorted to a supervised machine-learning approach, in which a random forest classifier was trained to infer the underlying locomotor condition based on alternative characterizations of the spatial organization of each individual map.

**Fig. 5:**
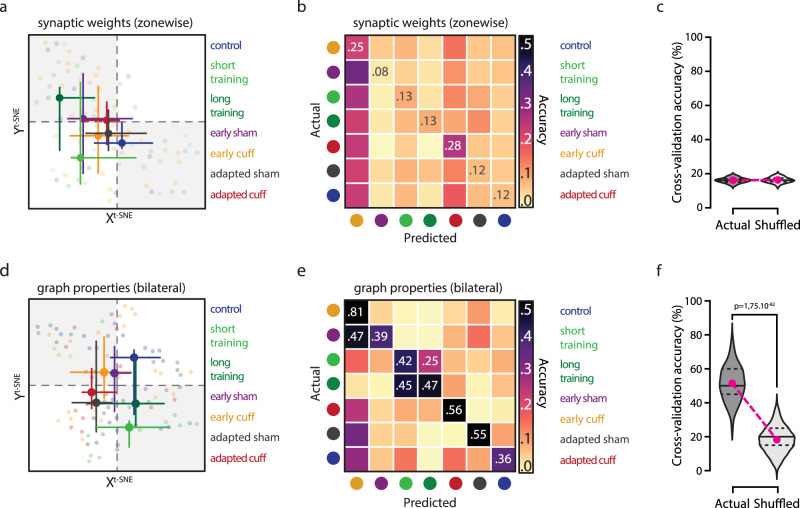
Graph features can predict locomotor conditions. **a** Dimensionally-reduced representation of synaptic connectivity maps, described in terms of averaged synaptic weights. Individual synaptic maps were parameterized as an eight-dimensional vector with entries given by the normalized, average synaptic weight in each microzone (Supplementary Fig. 1). These vectors were visualized in a 2D space via t-SNE, a non-linear dimensional reduction algorithm. Individual dots represent the position of each map in the projection space. Dots are colored according to the groups. Bigger dots represent the median of map positions for each group, and error bars the dispersion (interquartile range). *n* (maps/mice) control, 14/9; short training, 13/7; long training, 11/6; early sham, 11/7; early cuff, 25/13; adapted sham, 14/9; adapted cuff, 17/8. **b** Classification of synaptic maps based on synaptic weights. Map vectors described in **a** were maps used to train a supervised random forest classifier (*n* trial = 100). Accuracy of classification is displayed via a confusion matrix, the entries of which provide probabilities of correct classification (diagonal entries) or of misclassification (off-diagonal entries). **c** Average global accuracy of the trials performed with the random forest classifier shown in **b** for actual and shuffled labels (i.e., chance-level). Mean ± SD; 0.161 ± 0.01, *n* (actual) = 100; 0.163 ± 0.01, *n* (shuffle) = 100. Magenta dot, average; dashed-black line, interquartile range; solid black line, median. **d** Alternative t-SNE projection of synaptic maps reparametrized using graph properties (i.e., modularity index, ipsi- and contralateral assortativity, module degree *z*-score and participation coefficient; *n* (maps/mice) same as above**. e** Classification of synaptic maps based on bilateral graph properties using a random forest classifier (*n* trial = 100). Same representation as in **b. f** Average global accuracy of the trials performed with the random forest classifier shown in **e** for actual and shuffled labels (i.e., chance-level). Mean ± SD; 0.51 ± 0.1, *n* (actual) = 100; 0.18 ± 0.07, *n* (shuffle) = 100. Two-tailed MWU, *p*-value in graph. Magenta dot, average; dashed-black line, interquartile range; solid black line, median. Source data are provided as a Source Data file.

In a first attempt, we described each map’s organization through eight-dimensional vectors whose entries were given by synaptic weights averaged over the eight structural microzones (*g*_weights_zonewise_(*M*), zone-wise synaptic weights in Fig. 5; see Supplementary Fig. 1). Figure 5a shows a bi-dimensional visualization of the distributions of single-map vectors in our dataset, obtained via a non-linear dimensionality reduction algorithm (t-Stochastic Neighbor Embedding technique, t-SNE, see Methods section) to visualize clusters of maps with similar synaptic weight profiles. When maps were labeled by conditions, 2D projections showed strong scattering and overlapping centers of gravity (i.e., group medians of t-SNE projection coordinates, Fig. 5a), indicating a poor separation of behavioral groups. This intuition was confirmed by the poor performance of random forest classifiers trained on these input vectors (Methods section), as revealed by a confusion matrix analysis (giving the probabilities of correct classification or of misclassification among the adaptive conditions, Fig. 5b). Cross-validated classification performance was poor and comparable to chance level (average accuracy for all conditions upon cross-validation trials: 0.16 ± 0.01 for actual labels vs 0.16 ± 0.01 for shuffled labels, Fig. 5c). Therefore, we could not extract sufficient information from synaptic weights distribution in microzones to identify connectivity maps from different locomotor conditions.

In a second attempt, we described the fine structuration of connectivity maps in terms of a set of graph-based metrics (Fig. 5e and Supplementary Fig. 8). We provided as input a seven-dimensional vector (*g*_bilateral_(*M*)) with the following entries: the modularity index for the whole map, and graph participation, degree z-score, and assortativity averaged separately over nodes associated to the ipsi- or the contralateral map sections (bilateral graph properties in Fig. 5d–f). Unlike zone-wise synaptic weights, graph metrics have a complex dependency on map details and thus transcend anatomical subdivisions. Yet, all considered properties (apart from modularity) are evaluated for each single node, so that a tie to anatomy can still be maintained. For all groups and for all features we found once again a large variability across different maps and specimens, preventing us from detecting any significant group-level differences of individual graph metrics between the different adaptive conditions and the control maps (Supplementary Fig. 9). Nevertheless, when considered collectively, graph metrics carried useful information for condition discrimination. A t-SNE projection based on these alternative vectors of graph-based metrics showed that the cloud of maps for the different conditions were now more displaced with respect to the control maps (Fig. 5d). Furthermore, adapted sham/early sham and adapted cuff/ early cuff clouds were shifted away from the cloud of the short/long training conditions. Correspondingly, our supervised machine learning classifier now yielded a cross-validated classification performance larger than with synaptic parameters as well above chance level (average accuracy: 0.51 ± 0.1 for actual labels vs 0.18 ± 0.07 for shuffled labels, Fig. 5e, f). Some confusion in classification was still observed between the short training and the long training classes and between the early cuff and the early sham classes. Nevertheless, the mean cross-validated accuracy was ≥36% for all subtypes compared to ~18% of chance level and could rise to values as large as ~81% for the early cuff group (Fig. 5e). We also considered alternative graph parameterizations: a coarse one, in which graph features were averaged globally (*g*_global_(*M*)), thus fully ignoring anatomy; and a second, finer one, in which graph features were averaged by microzones (*g*_zonewise_(*M*), Supplementary Fig. 8a; Methods section). Both these alternatives, however, yielded worse performance, suggesting that fully ignoring anatomy is detrimental, but that information relevant for classification is not localized within individual microzones. In any case, Fig. 5e, f suggests that condition-specific map reorganization can be captured by graph descriptors for each behavioral condition.

As shown by Supplementary Fig. 8b, we also constructed random forest classifiers to discriminate maps at different developmental stages (cf. Fig. 2). Maps from the control groups of both the datasets of Fig. 2 (developmental) and 4 (adaptive) were correctly classified in a common class by the classifier. This fact confirms that our approach robustly operates even when maps are (down-)sampled to the lower spatial resolution of Fig. 2.

### Individual connectivity maps reflect individual-specific behavioral features

**Fig. 6:**
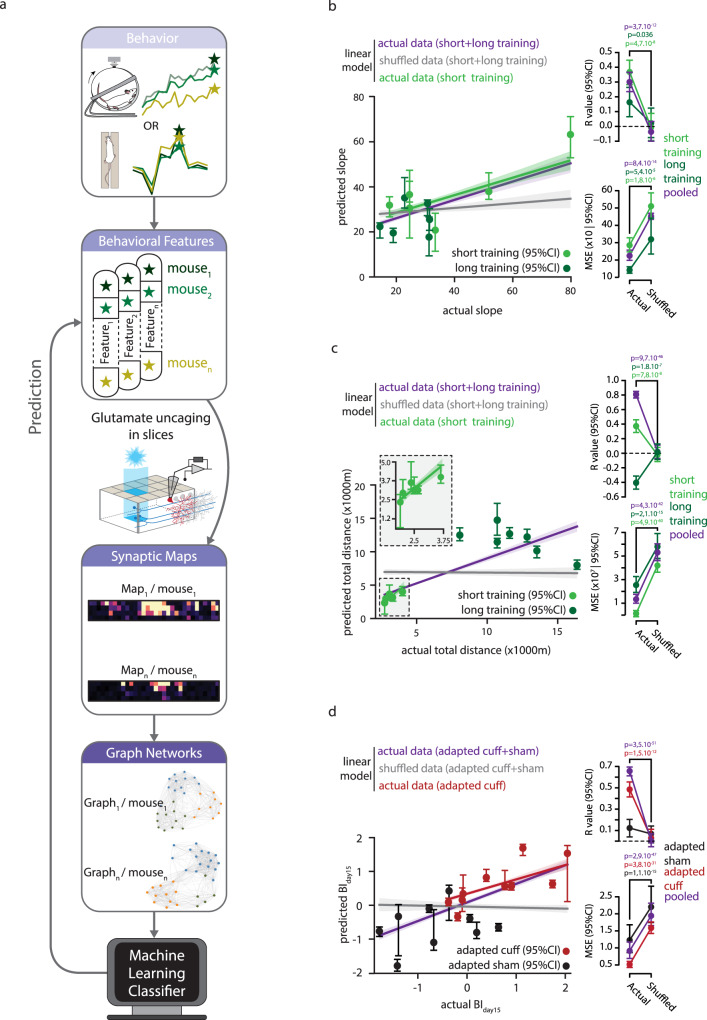
Graph-based prediction of individual performances. **a** Graphical illustration of the analytical pipeline to predict behavioral features from graph-based descriptions of synaptic maps. Locomotor performances in the wheel or in the force pressure corridor were summarized as behavioral features (see Fig. 3). Graph properties were computed from synaptic maps recorded in mice after completion of the behavioral tasks. Graph properties were then used as inputs to machine learning classifiers and GLM regression models in order to predict behavioral features at the level of single mice. **b**–**d** Behavioral feature predictions based on whole graph properties of synaptic maps using Generalized Linear Models (GLMs). In short (*n* = 13 maps/*N* = 7 mice) and long trained (*n* = 11/*N* = 6) animals, **b** the slope of performance and **c** the total traveled distance in the running wheel were considered as prediction targets. For adapted cuff (*n* = 17/*N* = 8) and sham (*n* = 14/*N* = 9) groups, **d** maximal impairment 15 days post surgeries was considered for prediction. For every prediction model, we show a scatter plot (with fitted line fits) of the actual vs the predicted values of the considered target feature. We quantified prediction performance using two metrics: Pearson correlation between actual and predicted values (*r*-values); and Mean Squared Error (MSE). The significance of performance (always in generalization conditions, i.e. performing predictions on synaptic maps not used for training) was assessed via comparison with chance-levels (from shuffled data, always shown in gray). We show tendency lines and performance indicators for the whole set of maps over which each model was fitted (purple) or restricted to specific subgroups (green or red hues). Center: average, error bars: 95% confidence intervals. *R*-value (slope, pooled, *n* = 200, mean ± SD): actual, 0.3 ± 0.47; shuffled, −0.04 ± 0.46; two-sided MWU, all *p*-values in graph. *R*-value (slope, short training, *n* = 200, mean ± SD): actual, 0.37 ± 0.63; shuffled, −0.01 ± 0.69; two-sided MWU. *R*-value (slope, long training, *n* = 200, mean ± SD): actual, 0.16 ± 0.7; shuffled, 0.02 ± 0.73; two-sided MWU. MSE (slope, pooled, *n* = 200, mean ± SD): actual, 224.5 ± 182.8; shuffled, 451.5 ± 539.2; two-sided MWU. MSE (slope, short training, *n* = 200, mean ± SD): actual, 285.3 ± 292.1; shuffled, 510.3 ± 504.4; two-sided MWU. MSE (slope, long training, *n* = 200, mean ± SD): actual, 142.03 ± 142.2; shuffled, 319.5 ± 749; two-sided MWU. R-value (total distance, pooled, *n* = 200, mean ± SD): actual, 0.81 ± 0.32; shuffled, 0.02 ± 0.57; two-sided MWU. *R*-value (total distance, short training, *n* = 200, mean ± SD): actual, 0.37 ± 0.63; shuffled, −0.02 ± 0.7; two-sided MWU. *R*-value (total distance, long training, *n* = 200, mean ± SD): actual, −0.41 ± 0.66; shuffled, 0.01 ± 0.77; two-sided MWU. MSE (total distance, pooled, *n* = 200, mean ± SD): actual, 133 × 10^5^ ± 234 × 10^5^; shuffled, 530 × 10^5^ ± 532 × 10^5^; two-sided MWU. MSE (total distance, short training, *n* = 200, mean ± SD): actual, 12 × 10^5^ ± 17 × 10^5^; shuffled, 419 × 10^5^ ± 420 × 10^5^; two-sided MWU. MSE (total distance, long training, *n* = 200, mean ± SD): actual, 253 × 10^5^ ± 483 × 10^5^; shuffled, 578 × 10^5^ ± 797 × 10^5^; two-sided MWU. R-value (BI_15_, pooled, *n* = 200, mean ± SD): actual, 0.66 ± 0.64; shuffled, 0.00 ± 0.52; two-sided MWU. R-value (BI_15_, adapted sham, *n* = 200, mean ± SD): actual, 0.12 ± 0.7; shuffled, 0.06 ± 0.7; two-sided MWU. *R*-value (BI_15_, adapted cuff, *n* = 200, mean ± SD): actual, 0.48 ± 0.62; shuffled, 0.03 ± 0.74; two-sided MWU. MSE (BI_15_, pooled, *n* = 200, mean ± SD): actual, 0.91 ± 2.25; shuffled, 1.94 ± 2.9; two-sided MWU. MSE (BI_15_, adapted cuff, *n* = 200, mean ± SD): actual, 0.52 ± 0.58; shuffled, 1.58 ± 1.52; two-sided MWU. MSE (BI_15_, adapted sham, *n* =  200, mean ± SD): actual, 1.23 ± 3.55; shuffled, 2.2 ± 4.82; two-sided MWU. Source data are provided as a Source Data file.

The large variability between maps may be due to experimental “noise” or reflect on the contrary fine levels of behavioral differences across individuals in normal or adaptive conditions. To investigate this hypothesis, we tried predicting individual-level specificities in adaptive locomotor behavior from graph descriptions of the global synaptic maps (*g*_global_(*M*)) and behavioral performance either on the wheel (short/long training) or on the force pressure corridor (adapted sham/cuff). We again used multi-dimensional vectors of graph-based map (*g*_global_(*M*)) descriptors as input and trained generalized linear models (GLMs) to predict individual-level behavior from spatial organization of their maps. The workflow of this analysis is described in Fig. 6a. Being now interested in individual-level differences, beyond group-level differences, we fitted jointly a GLM on all the individuals for which each given target behavioral feature had been measured. We thus pooled prior to GLM fitting: the short training and the long training groups, for both of which we measured slope and total distance in the wheel training (Fig. 3a); and the adapted cuff and adapted sham groups, for which we measured the BI time-course (Fig. 3c, d). Nevertheless, we took advantage of the GLM framework, to also include in the prediction model further interaction terms with the categorical label of the distinct groups being pooled (Methods). This allowed us to infer from data the possible existence of different graph-to-behavior relations within the groups being pooled, without assuming that these differences necessarily exist, as we implicitly did in the analyses of Fig. 5. We tested the efficacy of the GLM by testing the model on pooled data as well as the individual groups.

In Fig. 6b, c respectively we show a scatter plot of the actual values of total training distance/training slope and the corresponding values predicted by the GLM on the pooled, short and long training groups (Methods section). As revealed by superimposed trend lines, a correlation seems to hold between actual and predicted values, at the level of the pooled group, and sometimes even stronger when restricted to subgroups (Fig. 6c). We used two performance metrics to quantify the cross-validated performance of generalization prediction and avoid overfitting (i.e., prediction performed by models on data points for which they were not trained on, see Methods section): the Pearson correlation between actual and GLM-predicted values (*R*-value; top right insets *of* Fig. 6b, c); and the Mean Square Error of prediction (MSE; bottom right insets of Fig. 6b, c). Both these metrics were evaluated over the whole pooled short and long training groups or separately over the two groups. For the larger pooled dataset, both metrics were significantly better than chance expectations. *R*-values for pooled data reached 0.81 ± 0.04 (actual) vs 0.02 ± 0.08 (shuffled, Fig. 6c) for total distance and 0.3 ± 0.07 (actual) vs −0.04 ± 0.06 (shuffled, 95% CI, permutation testing, Fig. 6b) for motor training slope. The MSE for the pooled total distance data was 1.33 × 10^7^ ± 3.3 × 10^6^ (actual) vs 5.30 × 10^7^ ± 7.5 × 10^6^ (shuffled, Fig. 6c). Performances were even better when model was restricted to the short training group (Fig. 6c), while they were worse for the long training groups (Fig. 6c), possibly reflecting non-linear relations that the GLM framework cannot capture. The MSE for the slope followed a similar trend, for pooled data, 224.5 ± 24.7 (actual) vs 451.8 ± 75.6 (shuffled, Fig. 6b). The MSE are lower when only long training data is considered but worse when only short training data is considered (Fig. 6b). Even if behavior predictions are performed in terms of the multi-dimensional input vector of whole graph metrics, the model coefficients for the assortativity feature had a larger absolute value than all other features and a negative sign for the prediction of both slope and total distance of wheel training (Supplementary Fig. 10a, b). It means that maps with a lower assortativity, i.e., with a more variegated continuum of patch sizes (cf. Supplementary Fig. 4) tended to emerge in mice more eager to train in the wheel and/or in mice which were trained longer. The interaction coefficients with the class label in the GLM models of Fig. 6b, c were in most cases small, indicating that the relations between graph structure on one side and training behavior on the other are overall similar for the short and long training groups (especially for slope of training prediction, cf. Supplementary Fig. 10a).

In the case of the pooled adapted cuff and adapted sham groups, it was possible to follow the locomotor recovery on the force pressure corridor for weeks after surgery. We focused here on the BI_day15_ and AUC_early_ features (see Fig. 3c, d) as possible prediction targets monitoring behavioral recovery (see Supplementary Fig. 11 for AUC_early_ and AUC_late_ prediction). BI_day15_ is the behavioral feature with the larger dispersion of individual values. Indeed, we were able to significantly predict the evolution of locomotor unbalance monitored by the BI_day15_ component from graph description characterizations of functional connectivity maps (Fig. 6d), with a cross-validated R-value fit of 0.66 ± 0.04 (actual) vs 0.002 ± 0.06 (shuffled) for the pooled adapted cuff and sham groups. The R-value fit was lower, yet highly significant when the data were restricted to the adapted cuff group only, i.e 0.48 ± 0.07 (actual) vs 0.03 ± 0.09 (shuffled). The MSE for all the three groups of data, pooled, adapted cuff and sham were significantly lower than their shuffled counterparts (Fig. 6d). We were also able to predict to a certain extent the inter-individual differences in the early component AUC_early_. Although prediction performance was only marginally significant when mixing conditions (Supplementary Fig. 11), it improves up to 0.26 ± 0.11 (actual) vs 0.01 ± 0.12 (shuffled) when limiting the analysis uniquely to adapted cuff specimens. The prediction of the late component (AUC_late_) was also marginally significant (Supplementary Fig. 11). For the prediction of locomotor recovery features, GLM models had generally more homogeneous coefficients (Supplementary Fig. 10c, d), making it difficult to predict the effects of the variance in individual graph features on the behavioral features, as they could be modified by the variance in other features. Furthermore, interaction terms with the subgroup tended also to be greater, revealing a larger heterogeneity between the adapted sham/cuff groups than between the short/long training groups.

To conclude, the quality of prediction was heterogeneous across the different target behavioral features and subgroups (Supplementary Fig. 10c, d) as for some of the features, variability was smaller and thus inter-individual differences too fine to track. Therefore, we demonstrated that the graph features variability within each of the conditions, reflecting fine variations in the spatial structure of GC-to-PC connectivity maps, was not mere “noise” but carried information about the specific overtraining or post-traumatic recovery adaptation undergone by each individual.

## Discussion

We identified patchy GC functional synaptic connectivity maps to medial PCs as observed in our previous study^17^. These maps are acutely modified as a function of the locomotor context and during postnatal development. In this study, we took advantage of the zebrin band pattern to reliably compare and align connectivity maps between animals^39,43,44^. This framework defines specific boundaries of the cortical part of cerebellar modules, the microzones^42,60^. Indeed, many anatomical experiments tracing CF or MF projections in the cerebellar cortex showed that precerebellar inputs are well aligned with zebrin band boundaries^43,44,61^ (Supplementary Figs. 1 and 3). We found that synaptic maps re-organization across different locomotor contexts leads to an increase in the total GC-PC synaptic weights while on average the number of connected GC sites is conserved (Fig. 4a, b). Moreover, the more invasive was the condition (i.e., cuffed animals) the more important was the increase in synaptic weights and the full re-adaptation after surgery lead to a further increase in synaptic weights. Finally, a majority of GC layer sites undergo synaptic potentiation or awakening albeit some appear depressed as shown in ref. ^17^ (Fig. 4 and Supplementary Fig 6a). Although automatic locomotion does not involve cerebellar control^62^, adaptive locomotor behavior has been associated with the cerebellar cortex^24,63^. Therefore, our results strongly suggest that re-adapted locomotion is underpinned by strong synaptic plasticity in the cerebellar cortex re-organizing GC-PC synaptic maps at least for a month after an injury or learning of new motor skills. We acknowledge that other areas of the cerebellar cortex are certainly involved in these adaptations as recently suggested using mesoscale CF imaging^64,65^, but our recordings demonstrated that the vermal lobules III-IV encode at least partly this adaptive behavior. The patchy and context-specific reorganization of the GC-PC functional connectivity is also in agreement with the fractured somatotopy identified during in vivo micro mappings in the GC layer^47^. Remarkably, the characterization of the spatial organization of individual maps in terms of functional zones defined exclusively from graph descriptions and without any reference to anatomy retrieved functional zones which still bear information about zebrin band boundaries (Fig. 4, Supplementary Fig. 7). It confirms that anatomical structure contributes to shape the map reorganization under conditions involving locomotor adaptation. Indeed, based on the zebrin patterning, we showed that in control animals, medial PCs are connected by GCs integrating information from distal and proximal hindlimbs via bilateral lumbar and thoracic spinocerebellar (P2^+^/P1^−^_lateral_ and P1^−^/P1^+^_medial_) or ponto-cerebellar MFs (P1^+^ and P2^+^)^17,48,49,66–69^ (see Supplementary Fig. 1). Conversely, PCs are only sparsely connected by central P1^−^ GCs, which convey information from external cuneate nucleus and mediate forelimb proprioceptive inputs^48,66^. These results indicate that in natural locomotion medial PCs integrate both somatosensory and cerebral MF information from hindlimbs. In cuffed animals, denser connectivity maps including new GC layer areas receiving MFs from both forelimbs and hindlimbs were observed (e.g., in P2^−^ bands, Fig. 4e, Supplementary Figs. 1 and 6). Dense GC activity has been indeed observed in vivo when animals perform new complex task^70^. In addition, in all conditions except adapted sham and controls, hotspots of GC connections were observed at the middle of the ipsilateral P2 ^−^ band (~500 µm from midline). This part of the P2^−^ microzone receives information from both hindlimbs and forelimbs and targets hindlimb extensor muscles through the lateral vestibular nucleus^71,72^. We therefore postulate that in cuffed and wheel-trained animals, an intense reorganization of the coordination between forelimbs and hindlimbs is required for gait adaptation.

Many computational models suggest that the cerebellar microcircuits learn internal models of the body that are designed to predict an expected sensory feedback of a given command or simply update a motor command^18,20^. A change in the relationships between muscles or a modification of limb alternation during locomotion as we have induced  in our experiments should affect the workflow of computation involving internal models as also suggested in refs. ^5,24^. We provide strong evidence that PCs learn to adapt locomotor behavior by adjusting GC-PC connectivity maps in a context-dependent manner. In medial PCs, each context (e.g., gallop versus walk or sham versus cuff) leads to functional connections with a specific set of microzones through GCs. Moreover, multi-dimensional analysis of graph parameters (Figs. 5 and 6) defines a mathematical fingerprint of each connectivity map, which is predictive of individual behavioral features. The performance of the prediction may even be enhanced by further consideration of the GC-molecular layer interneuron-PC inhibitory pathway that could sharpen the graph representation of the synaptic maps. We therefore argue that the specific distribution of GC sites associated with medial PCs in the different behavioral conditions underlies locomotor adaptation and internal model adjustments.

Graph-based modules describe the complex functional structuration of the GC-PC connection. They were built by aggregating correlated columns of the GC layer in an unsupervised manner (Fig. 1 and Methods section), without preconceptions about the map’s spatial organization. As such, features of the graph community structure can account simultaneously for the degree of patchiness (high modularity index) and for the tight links between distant GC patches, thus being able to account for the complexity of MF somatotopy without presuming its role in the structuration of GC-PC maps. Graph networks are a powerful tool in the description of fractured functional microcircuit organization. For example, during postnatal development, we observed that between PND14 and PND18, synaptic maps were highly variable and median synaptic profiles close to a randomized, gaussian-like organization^73^. Graph parameters allowed us to quantify and highlight significant changes in the graph encoding of synaptic maps, notably modularity dropping to chance level values (Fig. 2f) indicating that modular structuration vanished at this stage before resuming to higher values in adulthood. Between PND14-18, mice not only start to walk and open their eyes^54–56^, several important molecular and morphological modifications also occur. For example, the GluN2C subunit incorporation in GC NMDA receptors leads to an enhancement of GC excitability^74^ and the regression of CF multi-innervation allows proper CF-dependent plasticity induction at the GC-PC synapses^75^. Therefore, we postulate that the PND14-PND18 period is a critical period allowing thorough reorganization of connectivity maps as observed in the visual cortex^76^.

Synaptic maps are non-randomly organized and their organization is highly and specifically adaptive (Figs. 4 and 5). It is therefore a question of utmost importance to understand how to appraise the spatial complexity of adaptive reorganization of these maps into a few quantitative metrics. Here, we translated synaptic functional connectivity maps to graph descriptions and described their topological features. The pertinence of our mapping was validated post-hoc by the quantitative superiority in the prediction that graph metrics confer with respect to other descriptions that were more tightly linked to anatomy (note dramatically reduced rates of class misclassification in Fig. 5d with respect to Fig. 5c). It is worth noting that graph metrics also reflect concrete properties of actual synaptic maps, even if they may seem more abstract than direct average measure of synaptic activation. Indeed, graph metrics are also computed exclusively in terms of synaptic map measurements (e.g., graph metrics on the level of microzones). However, they do not reflect uniquely local variations of connectivity (as the synaptic features used in Fig. 5a) but depend more generally on shorter- or longer-range correlations between connectivity at different distributed sites. Thus, the superior performance achieved by graph metrics may be inherent to the fact that they captured aspects of the spatial organization which are neither purely local (depending on a single map location), nor exclusively global (properties common to all map locations), as for example the redundancy of specific MF inputs in lobule III–V (Supplementary Fig. 1). Even when they are site-specific and heterogeneous across map locations, graph features describe properties of the coupling of these locations with many other locations. They thus reflect structure at many different and nested intermediate scales^77^, that overlap only imperfectly with traditional anatomical subdivisions (Supplementary Fig. 7) and that reveals a mixture of “segregation” and “integration”, two notions more commonly invoked in reference to neuroimaging data^78^. As a matter of fact, individual-specific adaptive behavior can be predicted by graph features even when averaged over all the sites in the map, thus fully ignoring anatomy (Fig. 6). At the same time, the discrimination between conditions is improved by using lateralized (*ipsi vs contra*) with respect to global graph metric averages but averaging to the level of individual microzones does not yield additional improvement (Fig. 5d vs Supplementary Fig. 8). It is thus likely that the number of available data for classifier training puts a limit to the amount of information that can be extracted without overtraining^79^ and that more precise characterizations could be still achieved by using larger datasets.

In conclusion, we postulate that classification and prediction of adaptive behavior is necessarily polythetic^80^, i.e. requires simultaneous monitoring of correlations between multiple graph features, because features taken one at a time have a stark variability and largely overlapping ranges between classes (see plots in Supplementary Fig. 9), as it is also observed for neuronal types^81^. Different adaptive conditions thus give rise to broad phenotypes of maps, in which connectivity is, nevertheless, not deterministically constrained, but preserves a large degree of freedom, exploitable to give rise to accurate individual behavioral adjustments. Graph-based features are only a first step toward the quantitative characterizations of the connectivity maps specificities at the single individual level. In the future, more powerful and general topological data analysis approaches^82^ may be used to capture the unique “shape” of each map and how the individualities of this shape reflect individual behavioral histories^83^.

## Methods

### Ethics and animals

All experiments were conducted in accordance with the guidelines of the Ministère de l’Education Supérieure et de la Recherche and the local ethical committee, the Comité Régional En Matière d’Expérimentation Animale de Strasbourg (CREMEAS) under the agreement no A67-2018-38 (delivered to the animal facility Chronobiotron, UMS3415, Université de Strasbourg). 83 AldoC-venus^45^ (^+/−^ or ^+/+^) male mice (Mus musculus) from PND9 to PND100 under CD1 background were used for this study. Mice were housed with all littermates and parents from birth to weaning age (PND21). Older mice from all experimental groups were housed by 3–5 littermates per cage, in conditions required to fulfill their ethogram, with nesting material, as well as food and water ad libitum in a 12/12 h light/dark cycle with constant hygrometry (45–50%) and temperature (21–22 °C; i.e., standard conditions described in Table 1).

### Surgical procedures for cuff and sham mice

Mice were anesthetized by inhalation of isoflurane (Verflurane, Virbac, France, 4% for induction then 1–2% for the surgery), then laid at rest on the left side of the body to expose the right hindlimb. A mix of lidocaïn/bupivacaïne (2 mg/kg each) was subcutaneously injected at the level of the upcoming incision. A 0.5 cm incision was made parallel to the femur to expose leg muscles. Muscles were gently separated using sterilized wooden sticks to expose the main branch of the sciatic nerve. The nerve was pulled out of the limb and a sterile 2 mm section of split PE-20 polyethylene tubing (cuff), 0.38 mm ID/1.09 mm OD, was wrapped around the nerve with the help of a pointed steel stick and a bulldog clamp. The nerve was then pushed back under the muscle fascia, and skin was sutured^59^. For sham mice, the exact same procedure was followed except that no cuff was implanted (i.e., the sciatic nerve was triturated, pulled out then pulled back under the muscle). After surgeries mice received a unique intraperitoneal injection of non-steroidal anti-inflammatory drug (Metacam, 2 mg/kg) and were left at rest for 24 h minimum before behavioral assessment. In cuffed animals, the plastic cuff remained around the sciatic nerve until the killing of the animals.

### Monitoring balance

To assess and quantify cuff-induced gait and balance impairments during locomotion, we built a force-sensor device. Mice were trained to voluntarily walk along an 80-cm-long corridor covered with two parallel strip-shaped force-sensors on each side of the corridor. The width of the corridor and the space between the two strips were adjusted to ensure that left and right limbs of CD1 adult mice are monitored by the corresponding strip (i.e., the force of the left limbs are measured exclusively by the left force sensor and vice versa). Force sensors (FSR, 10 × 622 mm each, FSR 408, Interlink Electronics, USA) are two-wire devices with a resistance that depends on applied force according to a logarithmic law, allowing simple force-to-voltage conversion when tied to a measuring resistor in a voltage divider. As mice enter the corridor, an IR-barrier connected to a Raspberry Pi microcomputer (Raspberry Pi Foundation, UK) triggers force and video acquisition. Force signals from the voltage-divider output were digitized at 15–20 kHz (NI USB 6211, National Instruments, USA). Force signals of the left and right side of the body were acquired simultaneously using WinWCP 4.2.2 freeware (John Dempster, SIPBS, University of Strathclyde, UK).

The balance index (BI; Fig. 3) corresponds to the log of the ratio of the integrated force signal from each side of the body. All recordings contained at least five consecutive strides, otherwise they were discarded. A unique datapoint was established as the average of minimum three trials for each mouse in each session. For further technical details (i.e., apparatus dimensions, scripts, and wiring diagrams) see https://github.com/ludo67100/cerebellarMaps/tree/main/Balance_supplementary.

In cuffed, sham, and control animals, the BI was determined before and after surgeries every 2–3 days for 1 month. A time-course was established, and we estimated the cumulative gait imbalance during the early days (AUC_early_ for the area under the curve; see Fig. 3), late phase (4–21 days after surgery; AUC_late_), and maximum imbalance (BI at day 15th after surgery).

### Locomotor training in a wheel (short and long training)

Trained mice had access to a running wheel for 1 h/day during 7 (short training) or 19 (long training) consecutive days. For each session, mice were placed in an individual cage equipped with a vertical, access-free running wheel. Locomotor activity was monitored using a piezoelectric sensor on the cage coupled to a magnet on the wheel to count the number of wheel turns during each session. We measured the total distance covered during individual sessions and during all the training periods of 7 or 19 days, and the slope of the training trajectory using linear regression analysis.

### DiI injections and measurement of parallel fiber extension

PND8-9 CD1 pups were placed in crushed ice for 2–3 min to be anesthetized. A small incision was rapidly made over the cerebellum and 0.5 to 1 µL fluorescent dye (Vybrant DiI cell labeling solution, ThermoFisher) was injected at the midline of lobules IV/V of the cerebellar cortex using a glass pipette and a pressure pump (Picospritzer III, Parker, USA). Location and depth of the injection were determined by eye using visual cues. After injection, the opened skin was closed with a drop of a biocompatible glue (Vetbond, 3 M, USA) and pups were placed on a heat pad a few minutes, then they returned to their homecage.

PND30 CD1 mice were anesthetized by inhalation of isoflurane (Verflurane, Virbac, France, 4% for induction then 1–2% for the surgery) and mounted on a stereotaxic frame (Model 68526, RWD Life Science). Body temperature was monitored using a rectal probe and maintained with a heating pad. A mix of lidocaïn/bupivacaïne (2 mg/kg each) was subcutaneously injected over the skull prior to incision, followed by an intraperitoneal injection of a non-steroidal anti-inflammatory drug (Metacam, 2 mg/kg). A parasagittal incision was made over the skull to expose lambda and bregma landmarks. The skull was cleaned using cotton sticks soaked in sterile 0.9% NaCl solution (saline). A 0.5 mm diameter hole was drilled at AP = −2 mm, ML = 0 (from Lambda) to expose vermal lobules IV/V. DiI was injected as described for pups. The skin was sutured after injection and animals were put back in their home cage

### Slice preparation for electrophysiology and photo-stimulation

Acute cerebellar slices were prepared from PND9–PND90 male CD1 ALDOC mice. PND12 to PND90 Mice were anesthetized by inhalation of isoflurane 4% (Verflurane, Virbac, France) and then killed by decapitation. PND9 and PND10 pups were sedated by hypothermia prior to decapitation. The cerebellum was rapidly dissected out and placed in ice-cold (≤4 °C) artificial cerebrospinal fluid (ACSF) continuously bubbled with carbogen (95% O2, 5% CO_2_), containing (in mM): NaCl (*120*), KCl (*3*), NaHCO_3_ (*26*), NaH_2_PO_4_ (*1.25*), CaCl_2_ (*2.5*), MgCl_2_ (*2*), glucose (*10*), and minocycline (0.00005) (Sigma- Aldrich, USA). In all, 300-µm-thick transverse acute cerebellar slices were then prepared with a vibratome (HM 650 V, Microm, Germany) in ice-cold (≤4 °C) *N*-methyl-d-glucamine (NMDG) based solution containing (in mM): NMDG (*93*), KCl (*2.5*), NaH_2_PO_4_ (*1, 2*), NaHCO_3_ (*30*), HEPES (*20*), Glucose (*25*), sodium ascorbate (*5*), Thiourea (*2*), sodium pyruvate (*3*), *N*-acetylcysteine (*1*), Kynurenic acid (*1*), MgSO_4_7H_2_O (*10*), and CaCl_2_.2H_2_O (*0.5*). After cutting, slices were maintained at 34 °C in bubbled ACSF for at least 45 min, then kept at room temperature until use (1–5 h).

### Patch-clamp recordings

Whole-cell patch-clamp recordings in voltage-clamp mode were obtained using a Multiclamp 700B amplifier (Molecular Devices, USA) and acquired with WinWCP 4.2.2 freeware (John Dempster, SIPBS, University of Strathclyde, UK). Patch pipettes (3–4 MΩ) were pulled from borosilicate capillaries using a gravitational puller (model PC12, Narishige, Japan). Series resistance was monitored and compensated (60–80% typically) in all experiments, and cells were held at −60 mV to isolate excitatory postsynaptic currents (EPSCs). The internal pipette solution contained (*in mM*): CsMeSO_4_ (*135*), NaCl (*6*), HEPES (*10*), MgATP (*4*), and Na_2_GTP (*0.4*). pH was adjusted to 7.3 with NaOH and osmolarity was set at 295–300 mOsm. Biocytin (Sigma Aldrich) or neurobiotin (Vector Laboratories, USA) were added (1 mg/ml each) for cell reconstruction. Voltages were not corrected for the liquid junction potential, which was calculated to be 9.8 mV (i.e., the membrane potential was 9.8 mV more hyperpolarized than reported). We accepted recordings for which the inward current at −60 mV did not exceed 1 nA. Synaptic currents in PCs were low pass filtered at 2.4–2.6 kHz, then sampled at 20–50 kHz. All cells were recorded in vermal lobules III, IV, or V, up to 130 μm from the midline. All experiments were performed at room temperature using the same bubbled ACSF than for dissection with inhibition blocked. We systematically blocked NMDA, adenosine, CB_1_, GABA_A_, GABA_B_, and mGluR_1_ receptors to limit the modulation of EPSCs amplitude by activity-dependent activation of these receptors. They were respectively blocked using (*in mM*): D-AP5 (*0.05*) (Ascent Scientific, Abcam Inc), DPCPX (*0.0005*), AM-251 (*0.001*), picrotoxin (*0.1*), CGP-52432 (*0.001*), and JNJ-16259685 (*0.002*) (Tocris-Cookson, UK).

### Photo-stimulation

Uncaging (i.e., photolysis of caged glutamate) experiments were performed using RuBi-Glutamate (100 µM, Abcam, UK) perfused in the recording chamber in a closed circuit. In ALDOC mice^45^, Venus fluorescence allowed direct visualization of PCs in the P1^−^_medial_ zebrin band (Supplementary Fig. 1). A micromirror DMD device (Mosaïc, Andor Technology, Belfast, Ireland) mounted on an upright microscope (Olympus BX51, Japan) allowed systematic photo-stimulation (steady single pulses of 30 ms) with a high-power LED (460 nm,UHP, Prizmatix, Israel) through a ×40 immersion objective (N-achroplan, NA: 0.8, Zeiss, Germany; Fig. 1a). The developmental dataset was performed with an orthogonal grid of 32 sites (40 × 40 µm per site; low-resolution) while synaptic maps recorded after locomotor adaptation were obtained with an orthogonal grid containing 96 sites (20 × 20 µm per site, high-resolution). A single grid covers 320 µm of the GC layer in the mediolateral axis. One synaptic map is composed of 2–4 adjacent fields of view (yielding 64 to 128/192 to 384 sites in total for low/high resolution respectively) covering up to 1280 µm along the mediolateral axis (see Fig. 1). Two photostimulations of a given site were separated by 60 s minimum. A single site of the granular layer was photo-stimulated between 5 and 10 times in total (yielding 5–10 recordings for averaging and analysis, Supplementary Fig. 2). Based on previous experiments and studies about light scattering in biological tissue, we estimated that light penetration was limited to <100 μm in the deepness of the slice^84–86^. Considering a volume of 20 × 20 × 100 μm (4 × 10^−5 ^mm^3^) with a density of 1.92 × 10^6^ GCs per mm^3^ (as described in Harvey and Napper^87^), the photo stimulation of this area activates at least 4 × 10^−5^ × 1.92 × 10^6^ = 76.8 GCs. Conversely, the stimulation of a 40 × 40 × 100 μm area activates 2 × 10^−4^ × 1.92 × 10^6^ = 384 GCs. These values might be underestimated since dendrites from GCs located in adjacent sites can be activated.

### Immunohistochemistry

After recordings, the patch pipette was gently pulled out from the soma to close the membrane and the slice was immediately transferred from the recording chamber to a fixation solution composed of 4% paraformaldehyde (Electron Microscopy Sciences) in ACSF for a maximum of 24 h at 4 °C. Zebrin bands were identified using intrinsic Venus fluorescence. Recorded cells were labeled using Alexa 555-Streptavidin (Thermo-Fisher, 1/1000, 3 h at room temperature). Ipsi- and contralateral P1+, P1−, P2+, and P2− Zebrin bands and distance between the recorded PC and the midline (P1+) were measured in each experiment. In adult CD1 mice, Zebrin band lengths in lobule III/IV/V were (µm, average ± SD, *n* = 100): *P2- contralateral* 416.6 ± 70.72; *P2* + *contralateral* 71.56 ± 24.59; *P1- contralateral* 320.516 ± 62.94; *P1* + 34.63 ± 16.18; *P1- ipsilateral* 320.46 ± 60.54; *P2* + *ipsilateral* 69.75 ± 22.53; and *P2- ipsilateral* 438.04 ± 64.25. Recorded PCs were located at 52.42 ± 29.8 µm from the midline of lobules III/IV/V, corresponding to the cluster 1 defined in Valera et al.^17^.

### Data processing and analysis

Data processing and analysis of synaptic maps and graph parameters were performed with customized scripts and routines written in Python 3.6 with the following packages: Pandas 1.3, Scipy 1.6, statsmodels 0.12, Sci-kit learn 0.24, Numpy 1.19, Neo 0. 10^88^, Orange 2.7^89^, bctpy 0.5.2. Plots were generated with Matplotlib 3.4 and/or Seaborn 0.11. Python-based code is available in the following repository: https://github.com/ludo67100/CereballarMaps-GraphProperties.

### Reconstruction of synaptic maps and profiles

For each experiment, the absolute positions of the recorded PC and photo-stimulation sites were normalized to the size of the corresponding P1^−^ ipsilateral Zebrin band (% of P1^−^). Median profiles and average maps were therefore obtained from spatially aligned maps within a global referential defined by the Zebrin band patterning, highly conserved between individuals (Supplementary Figs. 1 and 3; see also Apps and Hawkes)^39^. Each GC site was photostimulated 5–10 times and EPSCs were averaged. EPSCs elicited by RuBi-glutamate uncaging were measured in a 200-ms time window from stimulation onset (*A*_stim_ in Supplementary Fig. 2). For comparison, spontaneous activity in the slice was measured on each averaged recording of the map as the minimum amplitude in a 200-ms window before or after the photostimulation (*A*_noise_ in Supplementary Fig. 2). Such a measure of spontaneous activity was performed at all sites, yielding the distribution of the synaptic noise (X in Supplementary Fig. 2). *Z*-scores of the synaptic amplitudes for each site were calculated as follow:

1 $${{{{{\rm{Z}}}}}} {{{\rm\scriptsize{score}}}}=\frac{\left(A-X\right)}{\sigma }$$

Where *A* = maximal evoked synaptic amplitude, *X* = average of the synaptic noise, *σ* =  standard deviation of the synaptic noise. A GC site having a *z*-score ≥3 corresponds to an input that is statistically superior to the background noise and elicit a current above ~15 pA in PCs, which corresponds to <2 connected GCs in the photostimulated area containing tens to hundreds of GCs^16^. *Z*-scores were then used to threshold synaptic maps (Fig. 1b and Supplementary Fig. 3). For each GCL column, we used the maximum z score value of the column to define a projected profile along the mediolateral axis (Fig. 1b and Supplementary Fig. 2).

### Median synaptic profiles, cumulative synaptic weights, and averaged synaptic maps

We built the median GC-PC synaptic profiles and the averaged synaptic maps by pooling individual profiles and maps from each group. Individual synaptic profiles (*described above*) were interpolated and convolved with a triangular kernel (half-width = 9 or 18 µm for high- and low-resolution mappings respectively, see Supplementary Fig. 3a), then the median synaptic profile was calculated. These synaptic profiles were summed along the mediolateral axis and then averaged to build cumulative synaptic profiles shown in Fig. 4d, f. For averaged maps, individual maps and corresponding positional arrays were concatenated in separate vectors. The positional vector (containing relative positions) was sorted in ascending order along the mediolateral axis (from contralateral to ipsilateral positions in the GC layer) and the exact same sorting rule was applied in parallel to the map-based vector (containing synaptic amplitudes; Supplementary Fig. 3d). The resulting spatially sorted meta-map vector was then divided in 30 (high-resolution) or 60 (low-resolution) µm-wide bins for averaging. After averaging, and for visual purpose only, the maps were smoothed with a Whittaker-Shanon function.

### Maps down sampling

In order to compare maps from the development data set (low resolution) and the maps from control locomotion data set (high resolution) using graph-parameters (see below), high-resolution control maps were downsampled. This was done by convolving the high-resolution maps with a 2 × 2 box kernel and then downsampling it by (2, 2).

### Graph properties

#### Construction of the graph representation of GC-PC maps

To quantitatively parameterize and therefore compare the spatial structure of synaptic maps, we introduced a mathematical translation into graph representations (see Fig. 1 and Supplementary Fig. 4). The construction of the graph representation associated with each individual map relied on evaluating the spatial covariance matrix across the EPSC amplitudes profiles recorded for different map columns, since we note that patchiness in the map will tend to increase the overlap and then covariance between the profiles of columns occupied by a same set of patches. In each two-dimensional connectivity map, we considered the matrix *M*, whose *M*_*xy*_ entry denotes the EPSC amplitude recorded at the position coordinates (*x, y*). We then focused on the column vectors *M*_*x*_ of this matrix, giving the EPSC amplitude profile for a specific column at position *x*, and for every pair of positions *x* and *x′* along the scanned mediolateral axis we computed the normalized Pearson correlations *C*_*xx*′_ = corr(*M*_*x*_, *M*_*x*′_) between the connectivity profiles at the two considered positions. *C*_*xx*′_ values were then compiled in a correlation matrix C(M) (“Raw matrix” in Fig. 1c) that captured the patchy structure of the original map. *C(M)* can be considered as the adjacency matrix of a weighted undirected graph. The entries *C*_*xx*′_ thus become the strengths of links between graph nodes associated to the positions *x* and *x*′. The more similar are the connectivity profiles between two positions *x* and *x*′ and the stronger will be the weight *C*_*xx*′_ of the connection between them. A standard Louvain algorithm^52^ is then used to optimally partition graph nodes into non-overlapping communities such that total weight of within group edges are maximized and weight of between-group edges are minimized. A resorting of node labels according to the extracted community labels leads to the “Rearranged Graph Matrix” in Fig. 1c in which a block structure is better visible than before reordering of nodes. The resulting graph can be represented using a linear layout, in which the positions of nodes reflect exactly the ordering x of the original columns, or a “force-spring” embedding, in which nodes belonging to the same module are also positioned to be spatially closer between them, to emphasize the existence of modules in the graph visualization.

#### Graph-based features

All the absolute graph properties were calculated using the Brain Connectivity Toolbox for python (https://pypi.org/project/bctpy/) designed by Rubinov and Sporns^90^.

We characterized the GC-PC maps using graph properties such as:

(a) Modularity index - modularity index maximizes the number of within-group edges and minimizes the number of between-group edges using the Louvain community detection algorithm. It is a measure of how modular or “patchy” a graph is, i.e. higher the modularity index, the more distinct “patches” are in a graph. See Supplementary Fig. 4 for an intuitive accounting of how different map appearances translate into higher or lower modularity values. We use a variant of modularity index measure designed for weighted graphs given by the following equation^90,91^:

2 $${Q}^{w}=\frac{1}{{l}^{w}}{\sum }_{{ij}\in N}\left[{w}_{{ij}}-\frac{{k}_{i}^{w}{k}_{j}^{w}}{{l}^{w}}\right]{\delta }_{{m}_{i,}{m}_{j}}$$

Where *l*^*w*^ = total weight of the graph; *W*_*ij*_ = weight of the edge between node *i* and node *j*; ${k}_{i}^{w}$, ${k}_{j}^{w}$ = weighted degrees of node *i* and *j* respectively; ${\delta }_{{m}_{i,}{m}_{j}}=1$ if ${m}_{i}={m}_{j}$ and 0 otherwise; ${m}_{i,}{m}_{j}$ are the modules containing nodes *i* and *j* respectively.

(b) Module degree *z*-score (weighted) – is a measure of how relatively stronger or weaker is the connectivity of a node within a given module. It can be seen, in this sense, as a form of “degree centrality”, restricted to individual modules. A high average module degree *z*-score for the graph representation of a map indicates that patches in the map tend to be organized around a center, fading in a graded manner into the background which does not elicit stimulation responses in the considered PC. On the contrary, maps with lower average module degree *z*-score have internally homogeneous patches with sharper edges (cf. specific examples and pedagogic cartoon in Supplementary Fig. 4c). The weighted module degree *z*-score is given by the following equation^90^:

3 $${z}_{i}^{w}=\frac{{k}_{i}^{w}\left({m}_{i}\right)-{\bar{k}}^{w}\left({m}_{i}\right)}{{\sigma }^{{k}^{w}\left({m}_{i}\right)}}$$

Where ${k}_{i}^{w}\left({m}_{i}\right)$= weighted degree of node *i* with links between *i* and all nodes in *m*_*i*_; *m*_*i*_ =  module containing the node *i*; ${\bar{k}}^{w}\left({m}_{i}\right)$ = mean of the within-module degree distribution; ${\sigma }^{{k}^{w}\left({m}_{i}\right)}$ = standard deviation of the within-module degree distribution.

(c) Participation coefficient (weighted) - measures how strongly does a node in a module connects to nodes of the other modules. The participation coefficient is close to 1 if the connections received by the node are uniformly distributed among all modules and close to 0 if it favors connections to the nodes within its own modules. A high participation coefficient for a graph indicates that patches in the map tend to be partially overlapping in their range or imperfectly separated, as islands in an archipelago linked by narrow “piers” (cf. specific examples and pedagogic cartoons in Supplementary Fig. 4d). The weighted participation coefficient is calculated as the equation^90^:

4 $${y}_{i}^{w}=1-{\sum }_{m\in M}{\left(\frac{{k}_{i}^{w}\left(m\right)}{{k}_{i}^{w}}\right)}^{2}$$

Where *M* = set of modules; ${k}_{i}^{w}\left(m\right)$ = weighted degree of links between node *i* and all nodes in module *m*; ${k}_{i}^{w}$= weighted degree of links between node *i* and all nodes.

(d) Local assortativity (weighted) - is a measure of the tendency of a node to connect to other nodes with the similar degree. A positive local assortativity coefficient indicates that nodes tend to link to other nodes with similar degrees (e.g., if hubs tend to connect to hubs). Note that the node degree of a site within a map patch measures how many other sites the response profile of the considered site is similar to and that it is thus an indirect measure of the patch size scale. Thus, a map displaying both smaller and large patches well separated between them would result in a graph with a large assortativity (cf. specific examples and pedagogic cartoons in Supplementary Fig. 4e). The weighted local assortativity is calculated with the following equation^90^:

5 $${r}^{w}=\frac{{l}^{-1}{\sum }_{\left(i,j\right)\in L}{w}_{{ij}}{k}_{i}^{w}{k}_{j}^{w}-{\left[{l}^{-1}{\sum }_{\left(i,j\right)\in L}\frac{1}{2}{w}_{{ij}}\left({k}_{i}^{w}+{k}_{j}^{w}\right)\right]}^{2}}{{l}^{-1}{\sum }_{\left(i,j\right)\in L}\frac{1}{2}{w}_{{ij}}\left({\left({k}_{i}^{w}\right)}^{2}+{\left({k}_{j}^{w}\right)}^{2}\right)-{\left[{l}^{-1}{\sum }_{\left(i,j\right)\in L}\frac{1}{2}{w}_{{ij}}\left({k}_{i}^{w}+{k}_{j}^{w}\right)\right]}^{2}}$$

Where *l* = total weight of all links in the network; *L* = set of links between pairs of nodes *i* and *j*; *w*_*ij*_ = weight of the link between node *i* and *j*; ${k}_{i}^{w}$= weighted degree of node *i*; ${k}_{j}^{w}$=  weighted degree of node *j*.

Negative entries in the adjacency matrix were interpreted as an absence of similarity between the response profiles of two columns. Since our graph representations intend to describe relations of similarity, we thus ignored negative entries, treating them as zero for the computation of all graph features. All these graph metrics are then summarized into multi-dimensional vectors providing a parameterization of the spatial structure of each individual map. Modularity index is a graph centric measure and hence we get one value per map/graph. Module degree *z*-scores, participation and assortativity metrics are local node centric measures i.e, we get a different value per each node of the graph. We can then average these node-wise values through different coarser or finer averaging schemes, notably extracting: a single whole map median (yielding a four-dimensional vector *g*_global_(*M*) for each map); two medians, evaluated separately over nodes on the ipsilateral and the contralateral sides of the maps (yielding thus a seven-dimensional vector *g*_bilateral_(*M*) for each map); and, finally, different median values for nodes within each distinct anatomical microzone (yielding an eight-dimensional vector *g*_zonewise_(*M*)).

#### Relative values for graph-based features

Absolute values of graph features do not have a straightforward meaning. Furthermore for small graphs as the ones considered here may strongly fluctuate in value depending on graph size. However, values can be reported in a more informative way, by expressing them as percent differences with respect to chance level expectations for their corresponding null graphs. We then computed normalized percent differences:

6 $$\triangle {{\mbox{\%}}}=\frac{\left({{{{{{\mathrm{Actual}}}}}}}-{{{{{{\mathrm{Null}}}}}}}\right)}{{{{{{{\mathrm{Null}}}}}}}}\times 100$$

where, Actual = graph features of the actual graphs, Null = median of the graph features evaluated over a suitable null-hypothesis graph ensemble. Various instances of null graphs were created by re-wiring the original graphs but preserving their degree distributions. The routine randmio_und_signed from bctpy was used to generate the null graphs. The medians of graph properties over the generated ensemble of null graphs (minimum ten instances per each individual map) were used for the calculation of the relative values of the graph features.

#### Dimensionality reduction using t-SNE

t-SNE is a non-linear dimensionality reduction technique that transforms vectors in a high dimensional metric space into vectors within a lower dimension metric space (usually 2 or 3 target dimensions for the sake of visualization) while ensuring that close points in the higher dimensional space are mapped to points which are still close in the lower dimensional space, whereas dissimilar points are mapped farther away. t-SNE projections were used to reduce the dimensionality for two kinds of data: (1) the zone wise distribution of synaptic weights (*g*_weights_zonewise_(*M*)) and (2) lateralized absolute values of graph properties (*g*_bilateral_(*M*)). The sklearn.manifold.TSNE function in python was used. Centroids of point clouds corresponding to maps obtained in similar locomotor adaptation conditions have been obtained by computing the median projected coordinates of all the maps in the considered point cloud.

#### Subtype Classification using random forest classifier

The absolute graph features of three different resolutions (*g*_global_(*M*), *g*_bilateral_(*M*), and *g*_zonewise_(*M*)) and zone wise synaptic weights (*g*_weights_zonewise_(*M*)) were used to train and test four random forest classifiers in order to classify the different locomotion contexts (control, short and long training, early cuff and sham, and adapted cuff and sham). A random forest classifier uses multiple decision trees and combines their results to enhance the classification performance. The classifier used 150 individual tree estimators per tree ensemble, each with a maximum depth of 30. The data was divided into 100 stratified trials, with training:testing ratio of 80:20%. Proportion of correctly and misclassified samples (evaluated as generalization performance, i.e., applying the classifier on the testing subset not used for training) were quantified in a confusion matrix and the accuracy was calculated as a percentage of samples that were correctly classified. To compare the accuracy score with chance level scores, random forests were also trained on shuffled data, using identical hyper parameters but by shuffling target prediction labels. The sklearn.ensemble.RandomForestClassifier function from the sklearn package in python was used. We also used an analogous procedure to train a classifier to discriminate maps from different developmental stages.

#### Generalized linear models

In order to check how well the behavioral features of individual mice are predicted from the graph features of individual maps, we trained and cross-validated generalized linear models for 100 trials with training:testing split of 75:25%. The accuracy of the predictions on testing data (once again, generalization performance) was quantified by calculating *r*-values of fit and mean squared error between predicted and actual data for all 100 cross validation trials. To check the comparison with the chance level prediction, GLM was also trained and tested for the shuffled values of behavioral features in the training data. The *r*-value and mean squared error distributions of actual and shuffled GLMs are shown for comparison. The GLM equation posed the behavioral feature as a dependent variable and the graph-based features as independent variables. The contingency of graph features on the animal group (adapted cuff/sham or short/long training) is encoded as a bias variable (±1, e.g., adapted cuff = 1, adapted sham = −1) that is multiplied with the graph features:

7 $${Y}_{i}={\beta }_{0}+{\sum }_{j\in N}{\beta }_{j}\;.\;{G}_{{ji}}+{\sum }_{j\in N}{\gamma }_{j}\;.\;{T}_{i}\;.\;{G}_{{ji}}+{\epsilon }_{i}$$

Where *I* = trial number; *Y*_*i*_ = behavioral feature for trial *i*; *β*_*0*_ = common intercept; *N* = set of all graph-based features; *β*_*j*_ = slope for graph feature *j*; *G*_*ji*_ = value for graph property *j* in trial *i*; *γ*_*j*_ = group specific slope for graph feature *j*; *T*_*i*_ = ±1, for the two groups adapted cuff/sham & short/long training); *ϵ*_*i*_ = error. The statsmodels.api.GLM routine from the python statsmodels package was used.

#### Correspondence between anatomy- and graph-based zones

A mutual information analysis was used to quantify the degree of similarity between subdivisions of maps into zones based on anatomy (structural zones) or based on graph representations (functional zones).We thus first assigned structural and functional labels to each map column position *x* in the following way (cf. Supplementary Fig. 7 for a cartoon). Structural labels of a position, denoted by latin letters (a,b,c…), always refer to specific anatomical microzones. We then used two alternative ways of defining functional zones. In the first definition, two positions along the 1D map were assigned to a same functional zone if: they belonged to the same graph module; and all the positions lying between them also belonged to the same graph module. Such definition guarantees that the resulting functional zones are always spatially connected ranges (as anatomical microzones). The functional labels of a position are given by greek letters with a progressive integer index (α_1_, α_2_, α_3_, …, β_1_, β_2_, …, γ_1_…). Each greek letter is associated to a different connectivity module in the modular partition of the graph representation. If the same module includes nodes associated to distant, non-contiguous positions then multiple functional zones are generated out of the same module, one for each spatially connected range (numbered by the progressive index). The second definition of functional zones was similar to the first one (positions are grouped in the same functional zone if belonging to the same graph module), however, we dropped the criterion of spatial connectedness of the resulting range. In this way the partition in functional zones mirrored exactly the one of the graph into modules, however, some of the generated functional zones could be made of spatially disconnected ranges. In this second definition, functional zones were simply labeled by the greek letters of the corresponding graph module, without index (α, β, γ …). In all cases, we computed MI between the structural and functional labelings of column positions as:

8 $$I\left(X{{{{{\rm{;}}}}}}\;Y\right)={\sum }_{x\in X,y\in Y}{p}_{\left(X,Y\right)}{\log }\frac{{p}_{\left(X,\;Y\right)}}{{p}_{X}{p}_{Y}}$$

where* X* and *Y* respectively represent the strings of structural and functional zone labels for each map. *p*(*x, y*), where *x* ∈ *X*, *y* ∈ *Y*, is joint probability distribution of *X* and *Y*, that quantifies the overlap between structural and functional zone labels, whereas *p*(*x*) and *p*(*y*) represent the marginal probability distribution of X and Y. Chance-level values of MI could be estimated as well, by shuffling structural microzone labels and re-evaluating MI. MI values, for both actual and shuffled zoning, were normalized by the largest entropy value, among the entropies of structural and functional zone labeling:

9 $$H\left(X\right)=-{\sum }_{x\in X}\;{p}_{(x)}{\log }\;{p}_{(x)}$$

10 $$H\left(Y\right)=-{\sum }_{y\in Y}\;{p}_{(y)}\,{\log }\;{p}_{(y)}$$

so that 0 ≤ MI/H ≤ 1, with a value of one denoting perfect overlap between structural and functional zoning.

#### Statistics

Only biological replicates were used for statistics. Statistics were performed on independent experiments (i.e. one PC cell = one map = one slice = one experiment, 1–3 slices per animals) except in Figs. 2a and 4a in which the distribution of EPSC size of individual connected GC sites were used. Normality and homoscedasticity of the distributions were assessed with Shapiro-Wilk and Levene’s tests respectively in order to determine if statistical comparisons should be computed with parametric or non-parametric tests. Univariate statistical tests (Kruskal–Wallis, KW; Mann–Whitney *U*, MWU; Kolmogorov–Smirnov, KS; Levenes; Shapiro-Wilk, SW and paired or independent *t*-tests) were performed with the corresponding functions from the Scipy package. Unless reported differently, alternative and method arguments were set to ‘two-tailed’ and ‘auto’. Correction of Kolmogorov–Smirnov *p*-values with Holm method was performed with statsmodels.stats.multitest.multipletests function. Repeated Measure ANOVA on activity in the wheel and multivariate analysis of the BI time course (one-way repeated MANOVA and post hoc ANOVA) were performed using the Real Statistics Resource Pack (release 6.8), Copyright (2013–2021), Charles Zaiontz, www.real-statistics.com. Permutation testing was done by multiple bootstraps^92^ where a permutation of indices was used to calculate *r*-values and MSE for actual and shuffled data.

### Reporting summary

Further information on research design is available in the Nature Research Reporting Summary linked to this article.

## Supplementary information

Supplementary Information

Reporting Summary

## Data Availability

Source data are provided with this paper. Raw data were depositied in a public repository and accessible at 10.5281/zenodo.5714670. Source data are provided with this paper.

## Source data

Source Data

## Code availability

Code was registered in a public repository at https://zenodo.org/record/5745022 and is also accessible at https://github.com/ludo67100/CereballarMaps-GraphProperties.
