# Supplementary material for: Cerebellar connectivity maps embody individual adaptive behavior in mice
Source: Nat Commun. 2022 Jan 31;13:580. doi: 10.1038/s41467-022-27984-8 (PMC8803868; doi:10.1038/s41467-022-27984-8)
Supplement: Supplementary file 1 — Supplementary Information [file 41467_2022_27984_MOESM1_ESM.pdf]

Supplementary information Spaeth, Bahuguna et al.

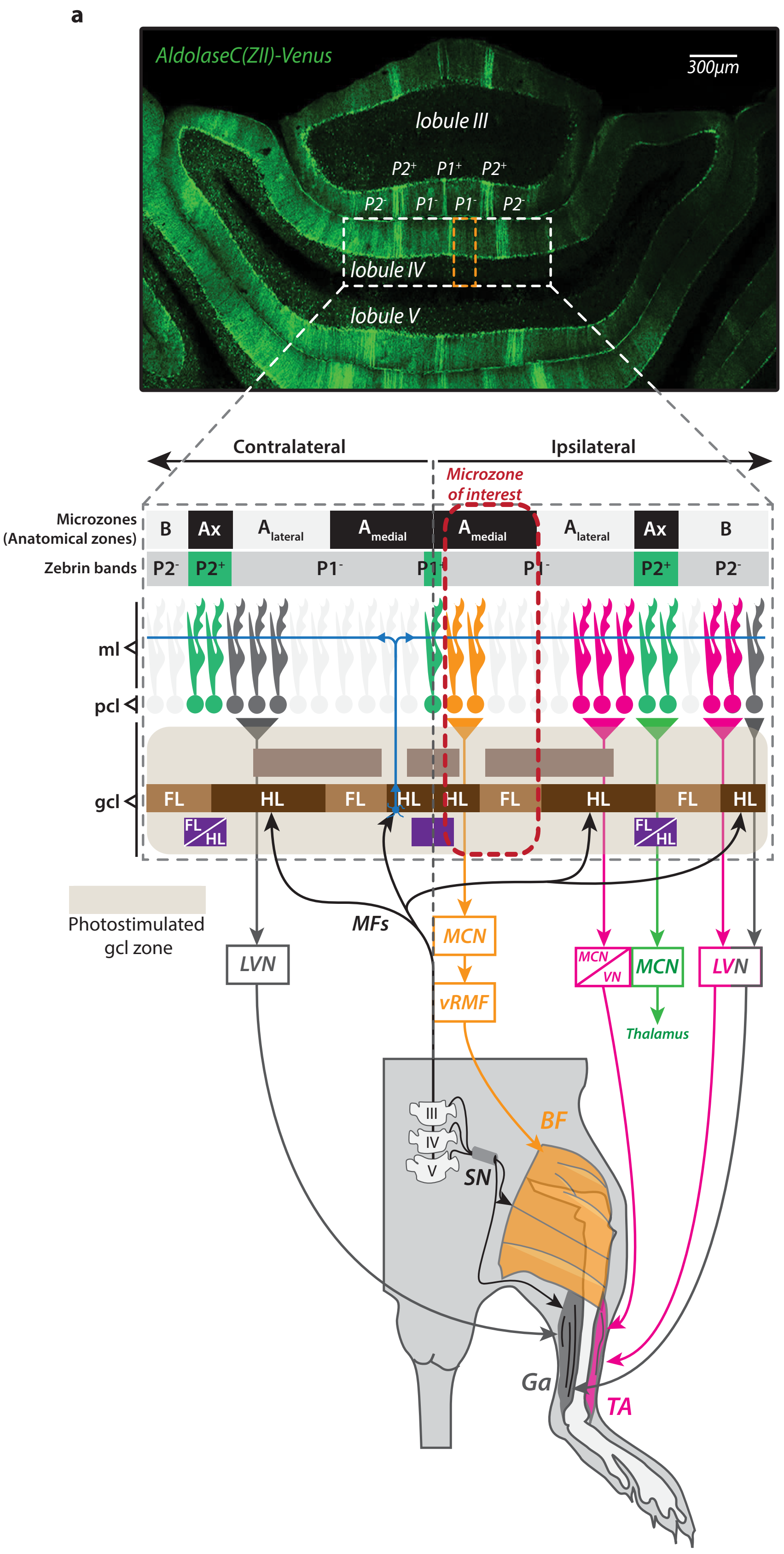

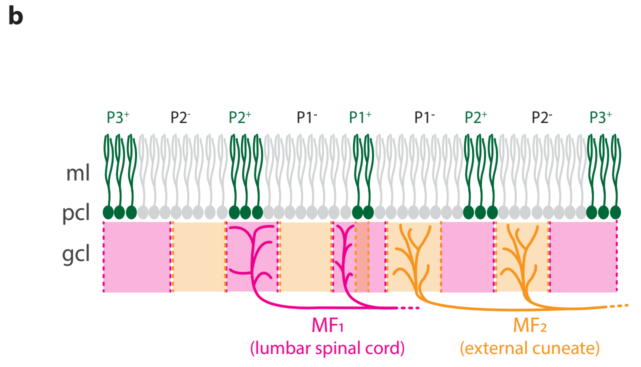

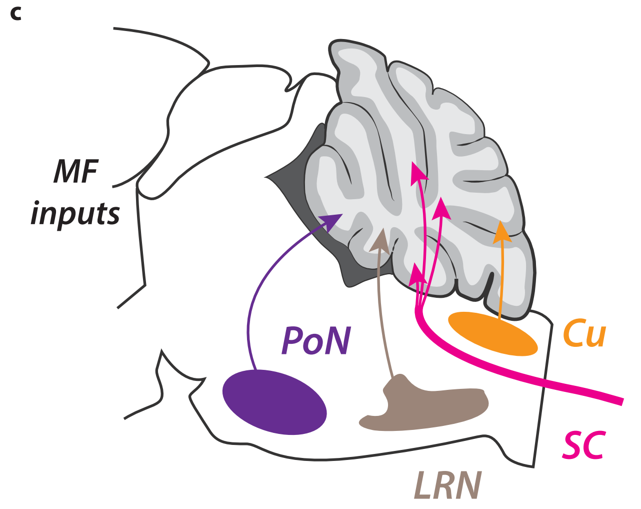

Supplementary Figure 1, legend next page →

Supplementary Fig. 1 | Input/output relationships and microzones in lobules III-V of the anterior vermis.

(a, top) Confocal image of a transverse cerebellar slice from an ALDOC-Venus mouse illustrating the Zebrin band patterning in the anterior vermis (n = 153 slices). The white-dashed box represents the photostimulation area in lobule IV of the cerebellar cortex. The orange-dashed box shows the microzone of interest (corresponding to the medial part of the A zone[43]), a 130 microns-wide parasagittal band starting at the midline where PCs were recorded. A microzone is defined as an area of the cerebellar cortex in which a group of PCs have similar climbing fiber receptive fields[60]. **(a, bottom)**, MF projections from pre-cerebellar nuclei to the anterior zones (bilateral A, AX, B) and corresponding PC output to other brain areas. Microzones target specific areas of the medial cerebellar (MCN) and vestibular nuclei (VN). The MCN projects to the ventromedial reticular formation while the LVN can project directly to the lumbar spinal cord controlling hindlimb muscles. Sensory information from hindlimb and forelimb muscles reaches the GC layer in different microzones via MF originating in L3 to L5 lumbar segments and the external cuneate nucleus, respectively. MFs relay on GCs (in blue) that send parallel fibers through the transverse plane of the molecular layer contacting hundreds of PCs in several microzones

(b) Diagram showing MF terminal fields in lobule III/IV of the cerebellar cortex. MFs originate in the lumbar level of the spinal cord (MF_1, magenta) or in the external cuneate nucleus (MF_2, orange)[48]. This diagram illustrates that MFs from a given region project to multiple locations of the GC layer.

(c) Schematic sagittal view of the mouse hindbrain illustrating pre-cerebellar nuclei from which MFs originate.

A/AX/B: anatomical microzones; **III/IV/V**: lumbar segments; **BF**: biceps femoris; **Cu**: Cuneate nucleus; **FL/HL**: forelimbs/hindlimbs; **Ga**: gastrocnemius; **GCs:** Granule cells; **gcl**: granule cell layer; **MCN**: medial cerebellar nuclei; **ml**: molecular layer; **VN**: vestibular nuclei; **LRN**: lateral reticular nucleus; **LVN**: lateral vestibular nuclei; **pcl**: Purkinje cell layer; P_n^+/ P_n^-: positive/negative Zebrin bands; **PoN**: pontine nuclei, **SC**: spinocerebellar tract; **SN**: sciatic nerve, **TA**: tibialis anterior; **vMRF**: ventromedial reticular formation.

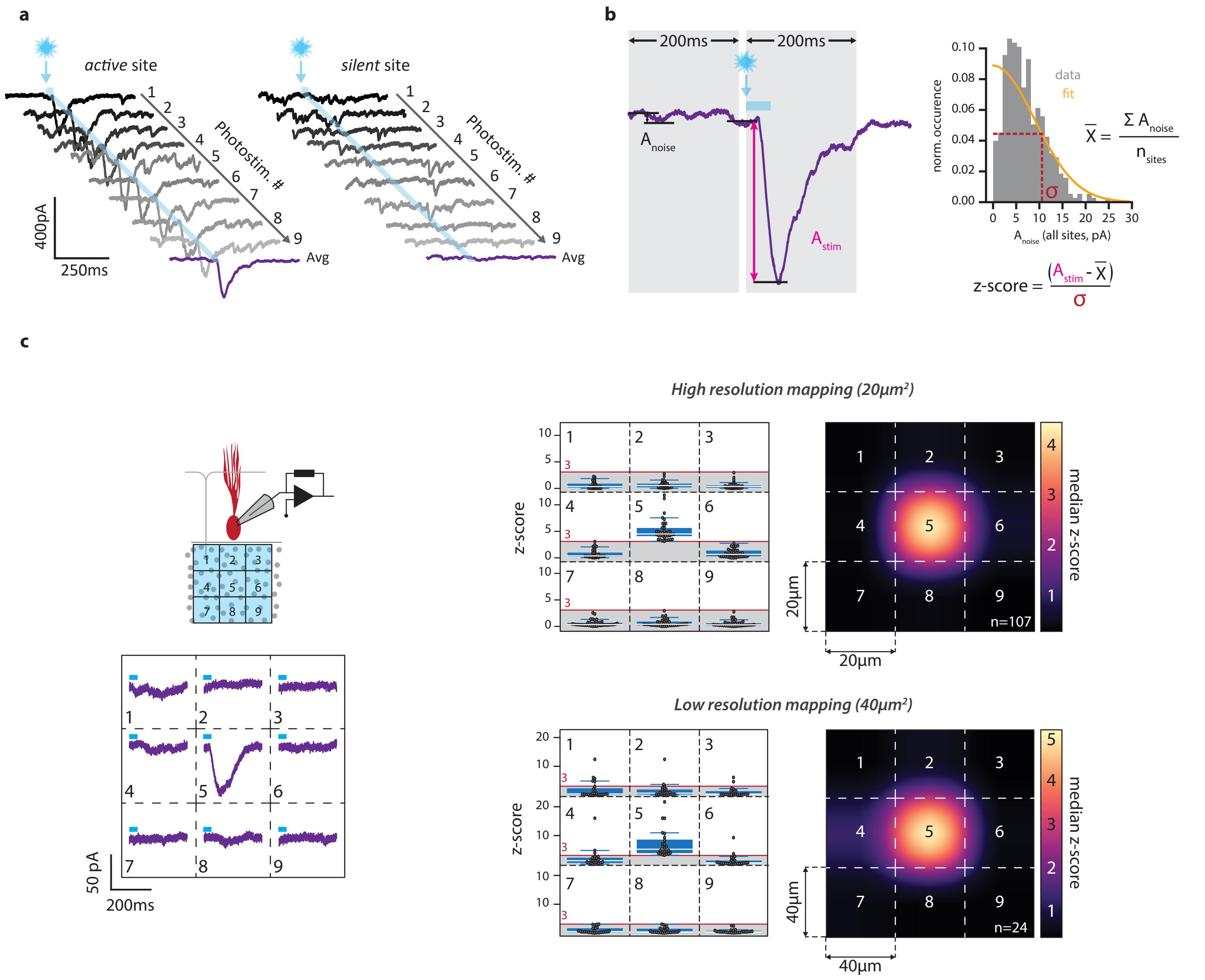

Supplementary Fig. 2 | Glutamate uncaging methods

(a) Glutamate uncaging is reproducible. Example of 9 consecutives traces recorded in a PC following photostimulation of an active (left) or a non-connected (right) GC layer site (20 x 20 µm, high resolution mapping, in shades of gray) and corresponding averaged EPSCs (in purple).

(b) Z score calculation. In each GC site, we measured (1) the amplitude of the averaged response (A_{stim}) in a 200 ms time-window following onset of light stimulation and (2) the amplitude of the averaged background noise (A_{noise} i.e. spontaneous GC activity). Distribution of A_{noise} values in a map (n = 128 for low resolution and n = 384 in high resolution mappings) was (1) averaged (X) and (2) fitted with a gaussian kernel to extract its standard deviation (σ). Z-scores of the averaged EPSC elicited by each GC site (1 response per uncaging site) was then calculated. GC sites eliciting averaged EPSCs with a z-score ≥ 3 were considered active as they correspond to more than 2 connected GCs in the photostimaulated volume (see Methods).

(c) Spatial accuracy of GC-PC connectivity maps. We assessed whether we could identify isolated GC-PC sites in connectivity maps at high and low resolution. 107 islands (i.e., an active site surrounded by silent sites) could be observed in high resolution (20 x 20µm) maps (n = 105 maps). Median of islands of the 107 GC sites yielded following z scores: site #1: 0.35 ± 0.49; site #2: 0.53 ± 0.49; site#3: 0.36 ± 0.34; site #4: 0.37 ± 38; site #5 (center): 4.50 ± 1.02; site #6: 0.66 ± 0.65; site #7: 0.35± 0.27; site #8: 0.41 ± 0.34; site #9: 0.3 ± 0.33. 24 islands could be identified in low resolution (40 x 40µm) maps (n = 48). Median of islands of the 24 GC sites yielded following z scores: site #1: 0.41 ± 0.45; site #2: 0.69 ± 0.84; site#3: 0.37 ± 0.37; site #4: 1.29 ± 1.27; site #5 (center): 5.25 ± 2.45; site #6: 0.51± 0.42; site #7: 0.5 ± 0.47; site #8: 0.39 ± 0.47; site #9: 0.41 ± 0.32. We postulate that RuBi-glutamate poorly diffuses outside individual illumination sites. Whisker bounds: minima/maxima, median, interquartile range. Source data are provided as a Source Data File.

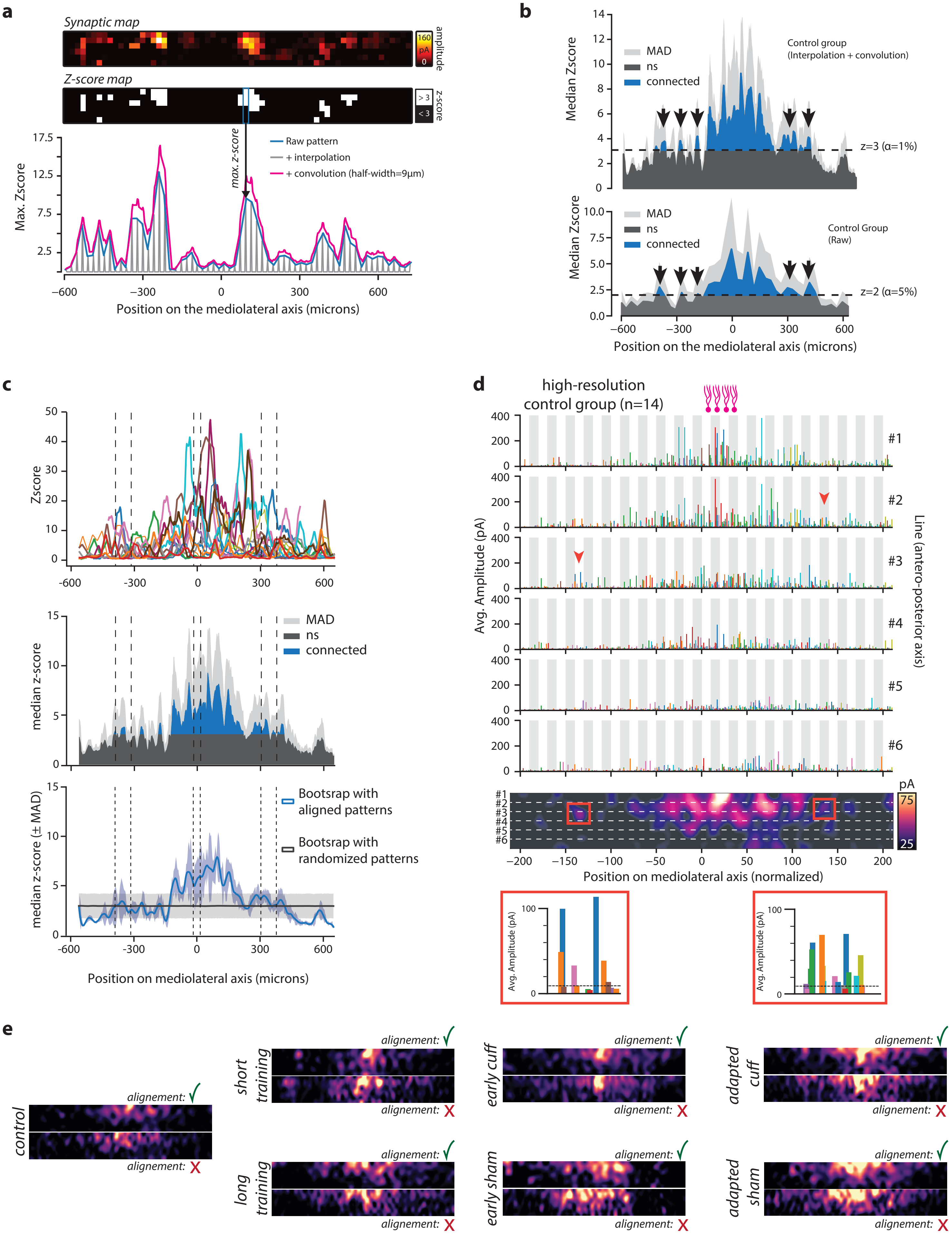

Supplementary Fig. 3, legend next page →

Supplementary Fig. 3 | Processing synaptic maps & profiles

(a) Synaptic maps (top) were first transformed into z-score maps. Synaptic profiles (1-dimensional vectors, bottom) are composed of the maximal z-score in each GC column along the mediolateral axis (blue curve). Prior to median calculation, individual synaptic profiles were interpolated (gray curve) then convolved with a triangular kernel (half-width = 9 microns, magenta curve).

(b) Pre-processing of individual profiles does not affect spatial accuracy of median calculation. **Top panel**, median z-score of the high-resolution control group after pre-processing of individual profiles (i.e., magenta curve in **a**). **Bottom panel**, median z-score of raw synaptic profiles (i.e., blue curve in **a**). Black arrows illustrate that the convolution does not affect the shape of the profiles.

(c) The spatial organization of the median synaptic profiles was not due to randomness. **Top panel**, superimposition of spatially aligned (alignment on Zebrin bands in each slice) individual synaptic profiles in control condition (n = 14 slices). **Middle panel**, median z-score profile along the mediolateral axis. **Bottom panel**, Median synaptic profiles were bootstrapped[92] 10000 times using the 14 profiles recorded in control condition. The blue curve shows the median from the spatially sorted profiles while the gray curve shows the median from randomized profiles (i.e., the position of each column was randomized along the mediolateral axis).

(d) Building averaged synaptic maps. **Top panel**, histogram of the mean synaptic amplitudes recorded in all animals from control conditions aligned to Zebrin bands (one color = one map, magenta PCs = average position of recorded PCs along the mediolateral axis). Since Zebrin bands are highly reproducible between animals, this method ensures a proper alignment between individual slices from different animals. Synaptic maps were built by binning values at each location and depth in the GC layer. White and gray columns represent the bins (10% $P1^-$ = 30µm) used for averaging. **Middle panel**, averaged synaptic map. Hotspots in red squares refer to the red arrow heads from the upper histogram. **Bottom panel**, inset of the two bins highlighted by the red arrow heads in the upper histogram. The dashed line represents the averaged value of synaptic noise.

(e) Effect of spatial alignment on map averaging. Top maps were aligned along the mediolateral axis prior to averaging. Bottom maps were stored in a common matrix, without any positional information prior to averaging.

Source data are provided as a Source Data file.

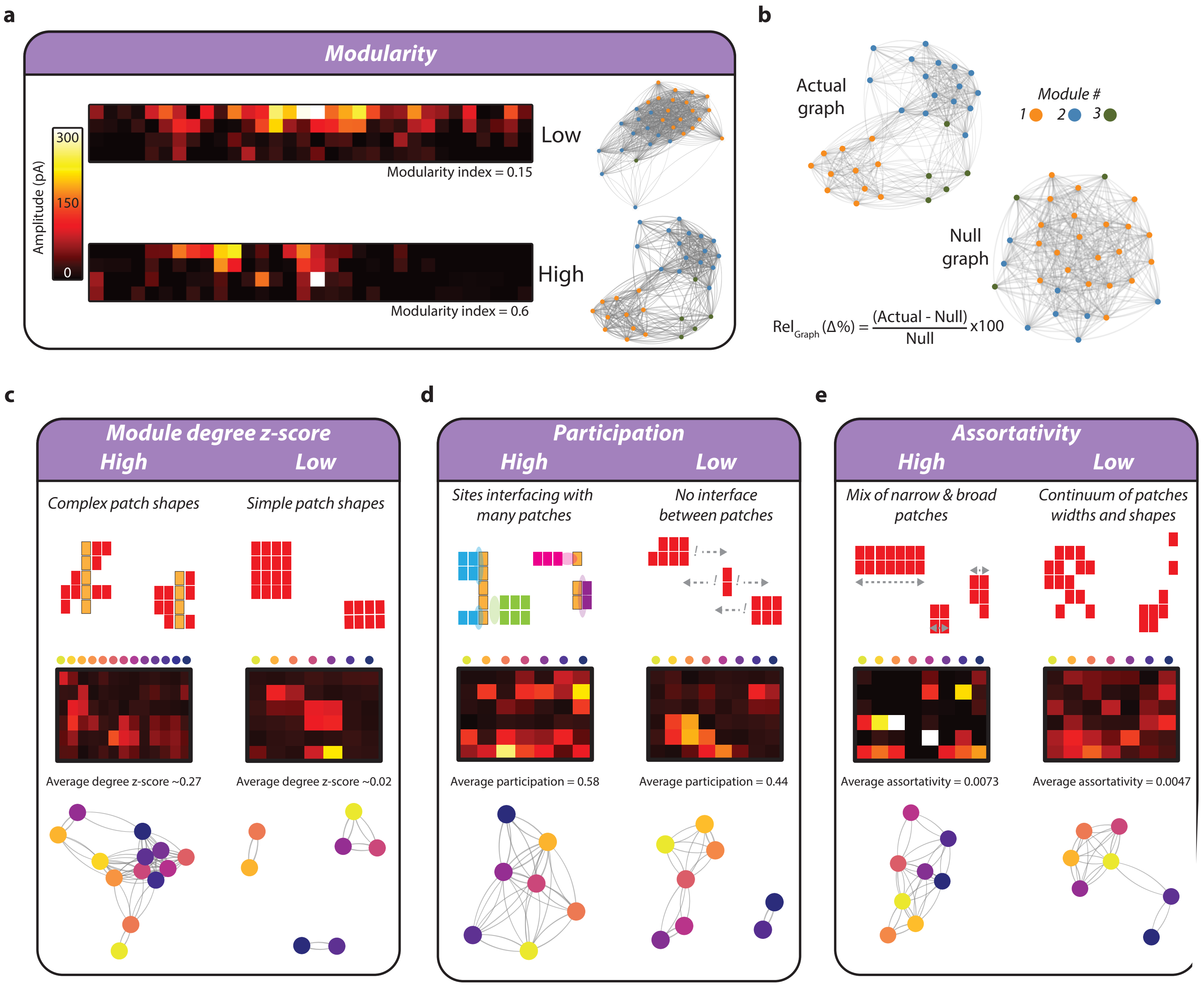

Supplementary Fig. 4 | Illustration of Graph Properties

Graph properties are mathematical descriptors of the spatial structure of synaptic maps. Although abstract in their formal definition, it is possible in some cases to intuitively understand how variations of graph properties correspond to specific changes in the patchy organization of the maps.

(a, left) Representative examples of synaptic maps showing low (top left) and high (bottom left) modularity indices. **Right**, corresponding spring-force graph representation.

(b) Illustration of actual graph vs an instance of a corresponding null-hypothesis graph, obtained by randomly rewiring edges while preserving the original degree distribution. Graph features evaluated on ensembles of null graphs provide as reference the chance level values against which the values measured in actual graph representations can be compared. Therefore, graph feature values shown in Fig. 2 & Supplementary Fig. 9 are normalized percent difference values, where Actual represents the actual graph properties of a map and Null represents the median of graph properties over an ensemble of at least 10 different null graph re-wirings.

(c-e) We show here selected examples of map sections illustrating how variations of the map patchiness result in variations of the module degree z-score (**c**), participation (**d**) and assortativity (**e**) graph metrics. For each feature we show first a pedagogic cartoon illustrating exaggerated patchy patterns producing higher or lower values of graph features (top). We then show portions of real synaptic maps (middle) including positions with relatively higher or lower values of the considered features and displaying patchy patterns reminiscent of the above exaggerated cartoons. Each position is encoded by a color gradient also used to color the corresponding nodes within a force-spring layout depiction of the graph representations of the shown map sections (bottom).

Supplementary note for Supplementary Fig. 4

To understand the logic ruling the construction of these pedagogic cartoons, it may be useful to remember that stronger or weaker graph connectivity between two nodes reflect larger or smaller overlap between the activation profiles (sequence of pixels eliciting or not PC response after photostimulation) along the corresponding map columns. In (**c**), patches with complex shapes will tend to be organized around a central axis (in yellow), which show large overlap with the profiles of many other columns occupied by the same patch. However other columns within the same patch may overlap between them at a weaker extent as the map shape is irregular. This will result in a variety of connectivity degrees among the nodes within the graph module corresponding to the considered patch. In other words, the module will contain strong connectivity hubs with higher degree z-score. On the contrary, patches with more regular shapes will give rise to more homogeneous overlap levels between the profiles of different member columns. In (**d**), certain columns (again in yellow) may display overlap with the profiles of other columns occupied by different patches, resulting in higher participation values (as the corresponding graph node will also have inter-modular connectivity beyond its own module). Lack of lateral overlap between patches on the contrary will tend to reduce participation. In (**e**), patchy patterns showing a mixture of broad and narrow patches will result in higher degree assortativity (i.e. high-degree nodes connected to high-degree nodes and low-degree nodes to low-degree nodes), as broad patches will give rise to larger sets of mutually interconnected nodes (hence all having large degree) and narrow patches to small sets of mutually interconnected nodes (hence, all having small degree). On the contrary, assortativity will be lower if there is a more graded continuum of possible patch sizes and shapes. As a concluding remark, let us note that situations akin to the pedagogic cartoons shown in panels (**c-e**) do give rise to upward or downward modulations of degree z-score, participation and assortativity. However, these metrics could be modulated by other types of patchy patterns as well, less prone to a simple and direct visualization. This is particularly true for graph modules composed of nodes which are not all spatially contiguous (i.e. graph modules extending over multiple functional zones, see Supplementary Fig. 7) and for which metric variations may be due to less intuitive configurations. Thus graph metrics can reflect the existence of specific patchy patterns, easy to seize, but can also capture geometry variations which would not be easy to describe trivially. Ultimately, graph metrics should be considered as a powerful characterization of map shape (and of differences between map shapes) “by numbers”, which is always rigorous and deterministic and which is able to capture perceivable differences in patchiness but also subtler and more general, combinatorially more complex geometry variations. c

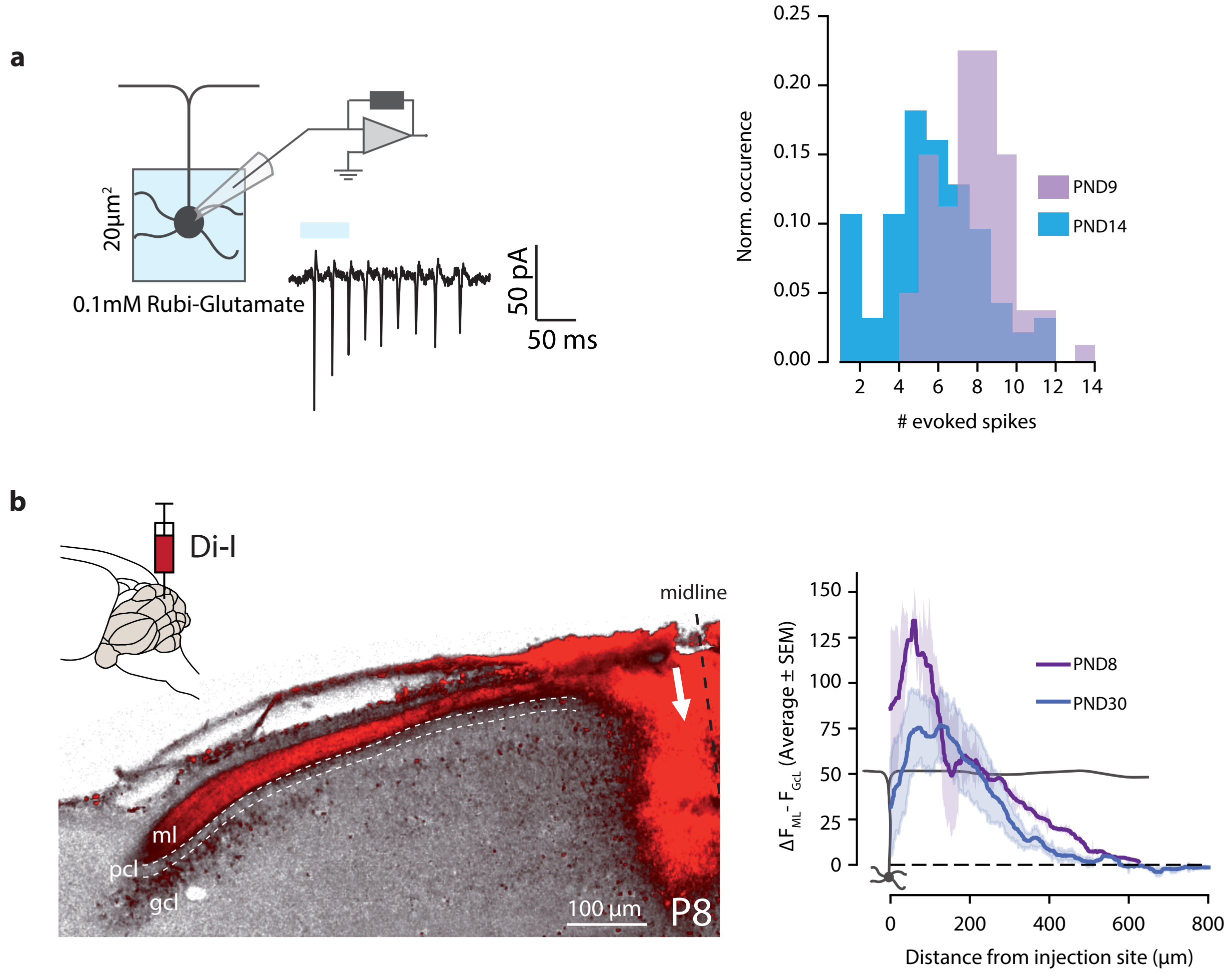

Supplementary Fig. 5 | GC excitability and Parallel Fiber length throughout postnatal development

(a) Measure of GC excitability before adulthood. Light-evoked action potentials in GCs were recorded in loose-cell attached (left panel, Methods) at PND9 and PND14. GCs (n = 4 in both conditions) were randomly selected in the vermal GC layer (lobules III, IV or V). Rubi-Glutamate uncaging (0.1mM, 20 x 20 µm, 30 ms steady-illumination) triggered 7.3±1.8 (mean ± SD) action potentials in P9 pups and 5.7 ± 2.3 (mean ± SD) action potentials in P14 mice (right panel, p=0.99, one-sided Mann Whitney U test). Reproducibility of GC firing patterns throughout consecutive photostimulation was previously assessed in Dorgans et al. (eLife, DOI: 10.7554/elife.41586).

(b) Measure of parallel fibers extension in pups and adult mice (observed in n = 2 mice in each group). Fluorescent Di-I was injected (Methods) at the midline of lobules III-V (left panel, injection site is shown with a white arrow). gcl: granule cell layer; ml: molecular layer; pcl: Purkinje cell layer. Fluorescence in ML and GCL were measured along the mediolateral axis from the injection site (right panel).

Source data are provided as a Source Data ile.

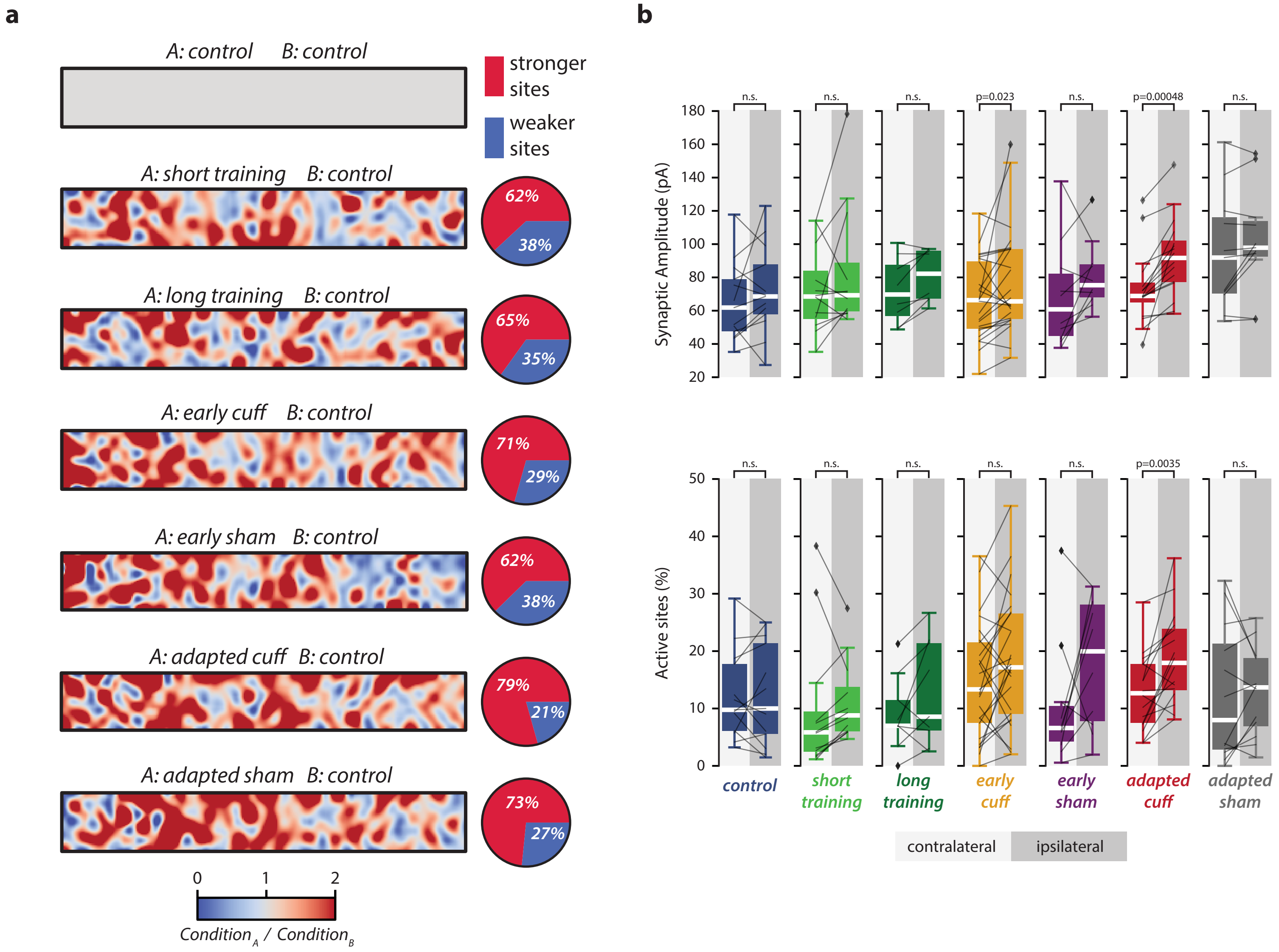

Supplementary Fig. 6 | Description of high-resolution synaptic maps

(a) Normalization of averaged synaptic maps from all groups to the averaged synaptic weights recorded in the control group. Red zones indicate where synaptic weights are, on average, higher than in the control condition. Conversely, blue zones show where synaptic weights are lower, on average, than in the control group. Pie charts show the proportion of stronger (red) and weaker (blue) regions for each comparison.
(b) Top panel, distributions of synaptic weights measured in ipsi- and contralateral sides of the synaptic maps in each condition (control, n = 14; short training, n = 13; long training, n = 11; early cuff, n = 25; early sham, n = 11; adapted cuff, n = 17; adapted sham, n = 14). Whisker bounds: minima/maxima, center: median, box: interquartile range. Diamond-shaped dots: outliers; n.s.: two-sided Wilcoxon signed-rank test, p>0.05, p-values in data source file; two-sided Wilcoxon signed-rank test, p- values < 0.05 in graph. **Bottom panel**, proportions of active sites measured in ipsi- and contralateral sides in each group. Whisker bounds: minima/maxima, center: median, box: interquartile range. Diamond-sh aped dots: outliers; n.s.: two-sided Wilcoxon signed-rank test, p>0.05, all p-values in Source Data. Two-sided Wilcoxon signed-rank test, p = 0.003483, W = 11.0.
Source data are provided as a Source Data file.

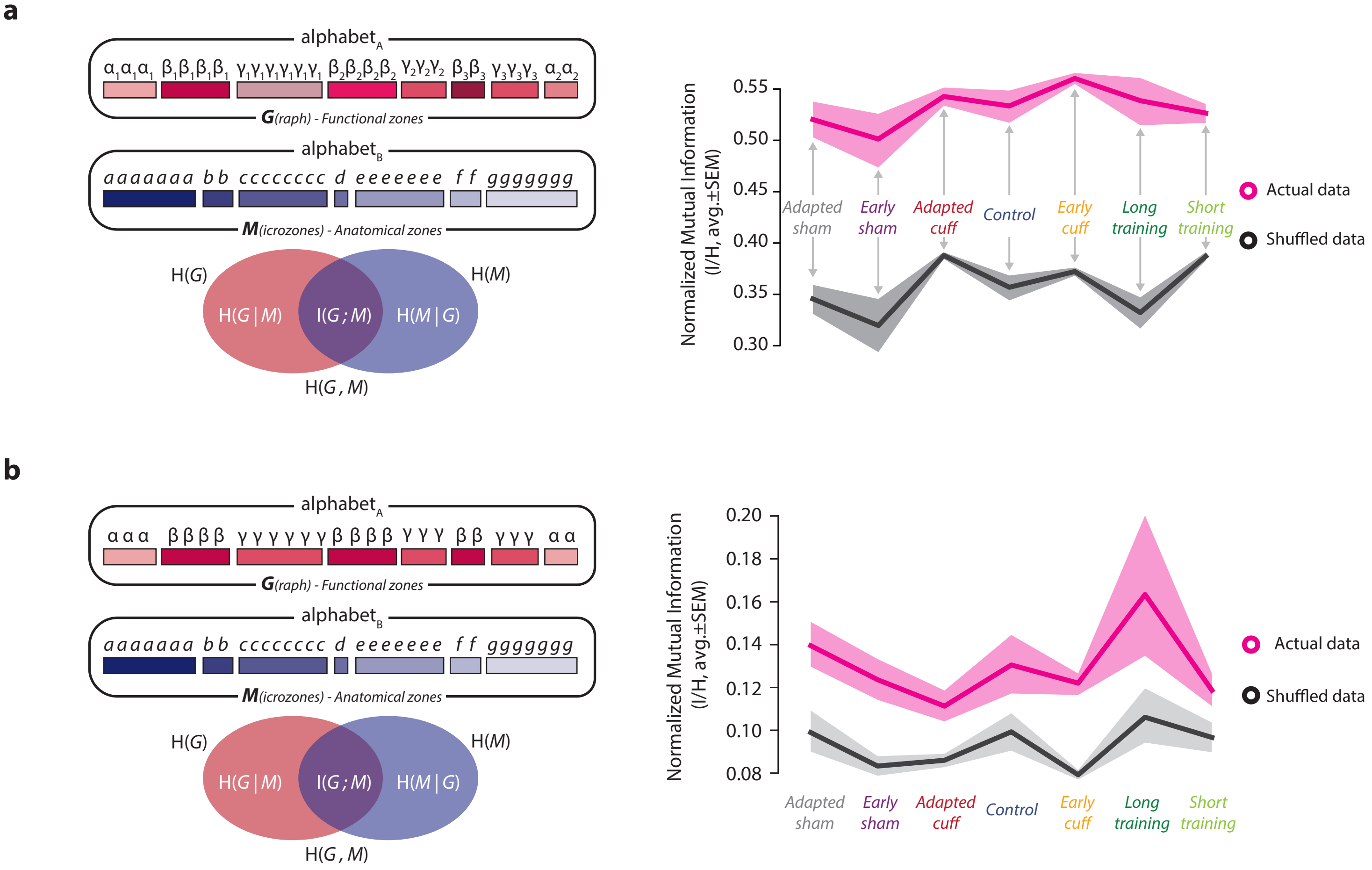

Supplementary Fig. 7 | Mutual information between subdivisions into microzones based on anatomy or graph structure

We define structural zones according to anatomical organization into microzones and functional zones derived from the modular partition of the graph representation. The overlap between structural and functional zones can thus be evaluated via a Mutual information analysis (normalized MI being always in the unit range and equal to one for perfect overlap) between zone labels and graph-based modules. As symbolized by a cartoon with intersecting Venn diagrams (**a**), MI captures the fraction of information that one subdivision into zones carries about the other one. The structural labels of a position are denoted by alphabets (a,b,c…), each corresponding to a specific anatomical microzone. We then used two alternative ways of defining functional zones (see cartoons on the **left** of panels **a-b**). (**a**) In the first definition of functional zones, two positions along the 1D map were assigned to a same functional zone if: they belong to the same graph module; and all the positions lying between them also belong to the same graph module. Such definition guarantees that the resulting functional zones are always spatially connected ranges (as anatomical microzones). The functional labels of a position are given by Greek letters with a progressive integer index (α_1, α_2, α_3, …, β_1, β_2, …, γ_1…). Each Greek letter is associated to a different connectivity module in the modular partition of the graph representation. If a same module includes nodes associated to distant, non-contiguous positions then multiple functional zones are generated out of a same module, one for each spatially connected range (numbered by the progressive index).
(**b**) The second definition of functional zones was similar to the first one (positions are grouped in a same functional zone if belonging to the same graph module), however we dropped the criterion of spatial connectedness of the resulting range. In this way the partition in functional zones mirrored exactly the one of the graph into modules, however some of the generated functional zones could be made of spatially disconnected ranges. In this second definition, functional zones are simply labeled by the Greek letters of the corresponding graph module, without index (α, β, γ, …). To the **right** of panels (**a**) and (**b**), we show the obtained values of normalized MI between structural and functional zones for all locomotor conditions (pink) and for both definitions of functional zones (average ± SEM). The MI for shuffled data (shuffling the microzones labels) are also shown for comparison (grey). For both alternative definitions of functional microzones, normalized MI is robustly above chance level for all conditions, suggesting that functional zones boundaries are significantly aligned with anatomical microzones, although not perfectly. Source data are provided as a Source Data file.

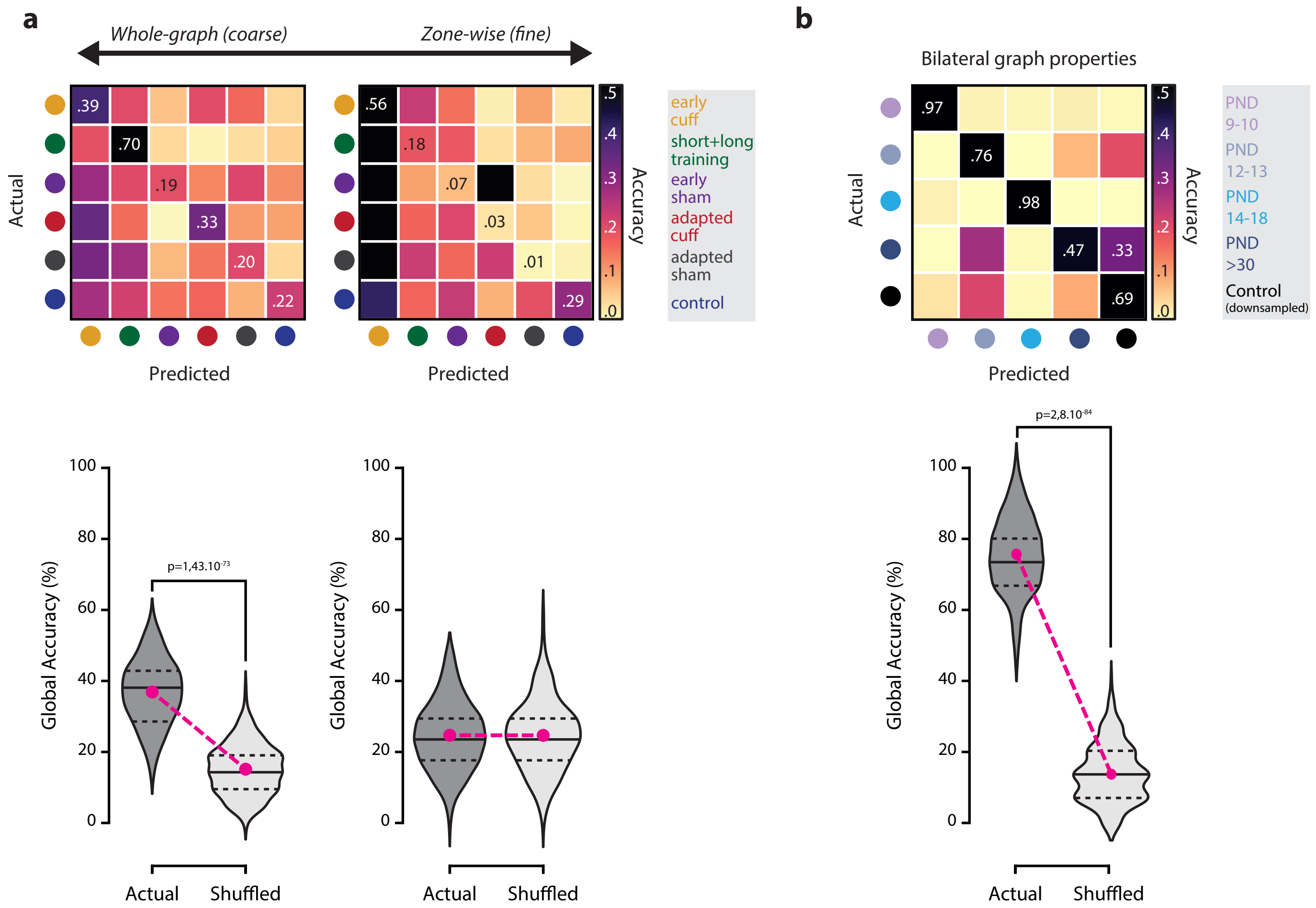

Supplementary Fig. 8 | Extended analyses with Random Forest classifiers

Additional classifiers for the discrimination of different map classes, based on alternative datasets or sets of input features.

(a) In figure 5, we performed group classification in terms of seven graph features, including whole graph modularity and lateralized averages of the other graph-features. We also tried classifying groups based on: (***left***) fewer graph features (*four*), replacing lateralized with whole map averages; and, (***right***) more graph features, replacing now lateralized with zone-wise averages (*8 zones*). In both these cases, the performance was worse than with bilateral graph properties shown in Figure 5e. These results suggest that accounting for anatomy carries additional information with respect to whole-map averaging. However, the amount of available data may not be enough to use input representations with too large dimensions (or structural microzones may not be the right zoning to use). Bottom violin plots show the average global accuracy of the 100 trials performed with the random forest classifier shown above, for actual and shuffled labels (i.e., chance-level). Magenta dot, average; dashed-black line, interquartile range; solid black line, median. Two-sided MWU test, w = 60411.0, p = $1.43.10^{-73}$. (**b**) We also constructed a random forest classifier based on the data of Figure 2, to discriminate developmental classes based on the same seven graph features of figure 5. Here however, the original map resolution was lower. Even in this case we could properly discriminate between groups well above chance level. Note that when attempting to classify down sampled control maps from the dataset of Figure 5 with this new classifier it was strongly confounded with the adult PND>30 control group for the developmental dataset of Figure 2. This indicates that the two control groups for the separate experiment carry an analogous structure of their graph representation and that this statistical equivalence can be detected even when operating at a lower (natural or artificially reduced) resolution. Bottom violin plots show the average global accuracy of the 100 trials performed with the random forest classifier shown above, for actual and shuffled labels (i.e., chance-level). Magenta dot, average; dashed-black line, interquartile range; solid black line, median. Two-sided MWU test, w = 62500.0, p = $2.82.10^{-84}$. Source data are provided as a Source Data file.

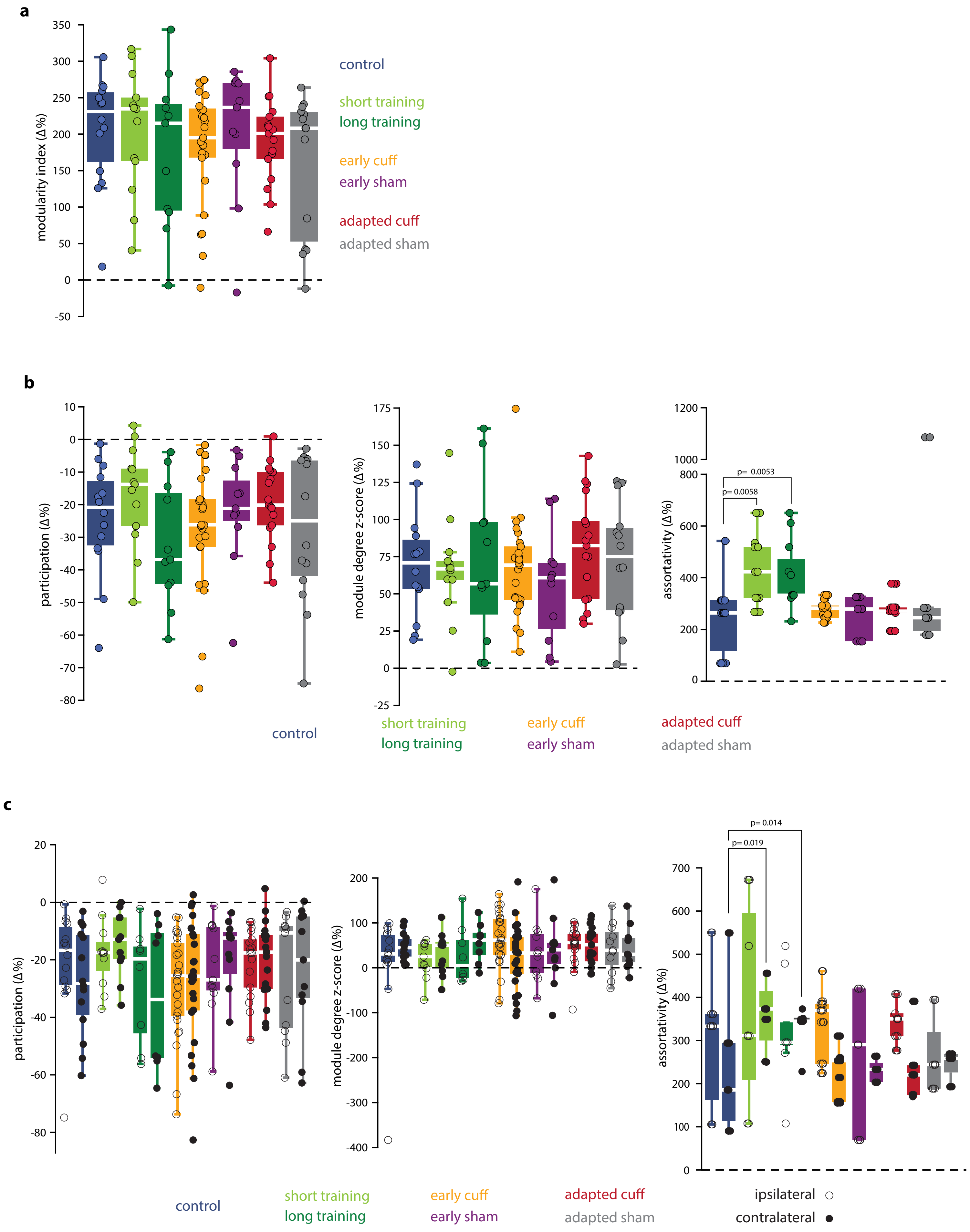

Supplementary Fig. 9, legend next page →

Supplementary Fig. 9 | Graph properties from high resolution dataset for adapted locomotor conditions

Boxplots report normalized percent differences of graph metrics with respect to chance level values for null-graphs (dashed lines at zero) for all locomotor conditions. The large dispersion denotes for all properties and conditions a large individual variability of maps within each class. We show first properties averaged, per each PC, over the whole map. In panel a-c: control, n (maps) = 14; short training, n = 13; long training, n = 11; early cuff, n = 25; early sham, n = 11; adapted cuff, n = 17; adapted sham, n = 14. Whisker bounds: minima/maxima, center: median, box: interquartile range.
(a) Modularity index; Kruskal-Wallis test, p=0.66. All p-values in data source file.
(b) Left panel: Participation coefficient; Kruskal-Wallis test, p=0.49. Middle panel: Module degree z-score; Kruskal-Wallis test, p=0.74. Right panel: Assortativity. Kruskal-Wallis test, p = 0.000609; Post-hoc Mann-Whitney U test (two-sided), p-values in graph. All p-values in Source Data.
(c) Same as (b), but lateralized graph properties, i.e. averaged separately over the ipsi- and contralateral sections of the map. The empty circles represent the ipsilateral graph properties and filled circles represent the contralateral graph properties. Kruskal-Wallis test between all conditions in Assortativity, p = 0.000531; Post-hoc Mann-Whitney U test (two-sided), p-values in graph. Whisker bounds: minima/maxima, center: median, box: interquartile range. All p-values in data source file. Source data are provided as a Source Data file.

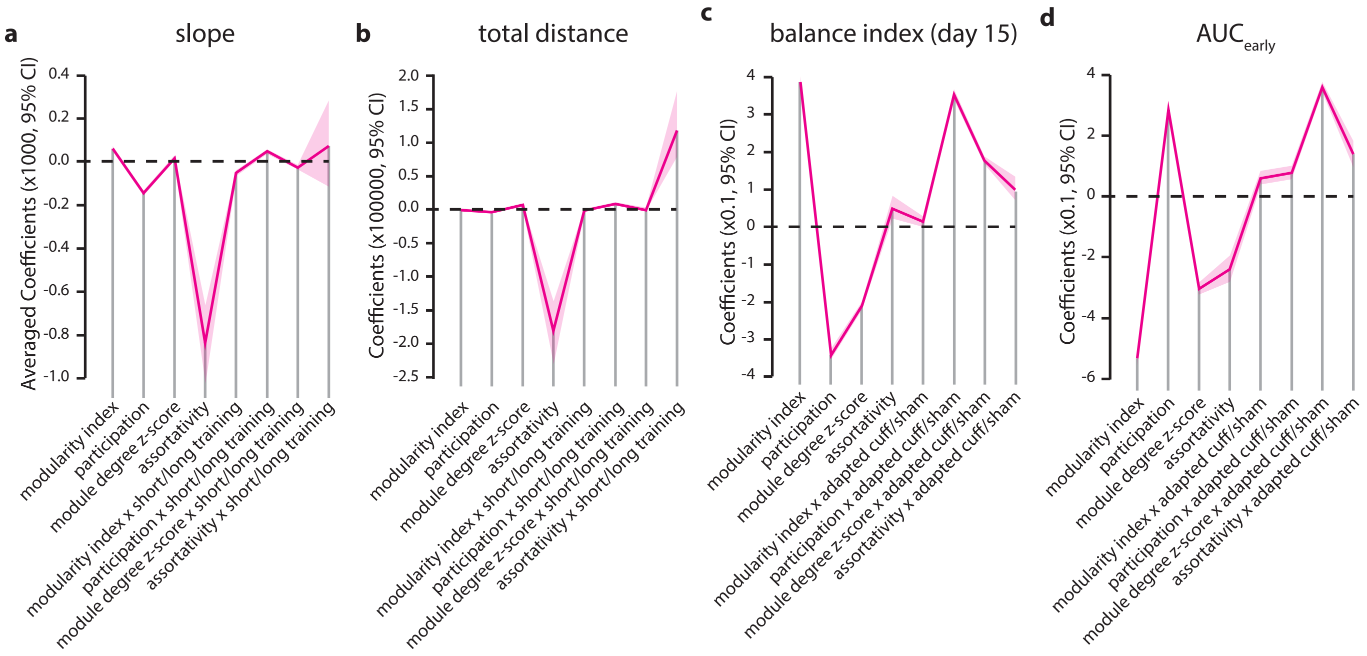

Supplementary Fig. 10 | Coefficients in GLMs

Signed coefficient distributions for the four GLMs shown in Figure 6b,c,d,e. The thick curve shows the mean, the shaded area represents the 95%CI and all the independent variables are shown on the x-axis. Please note, that the variables consist of the class independent terms (eg modularity_index) as well as the interaction terms (eg. modularity_index X short/long training in **(a)**). The variables that strongly modulate the behavioral features show a significant non-zero coefficient distribution (eg. assortativity in **(a)**). The sign of the coefficients indicates the direction of the influence on the behavioral feature (eg. an increase in assortativity is proportional to decrease in slope in **(a)**). The coefficient distribution profiles for **(a)** slope **(b)** total distance **(c)** balance index from day 15 **(d)** AUC_{early} . Source data are provided as a Source Data file.

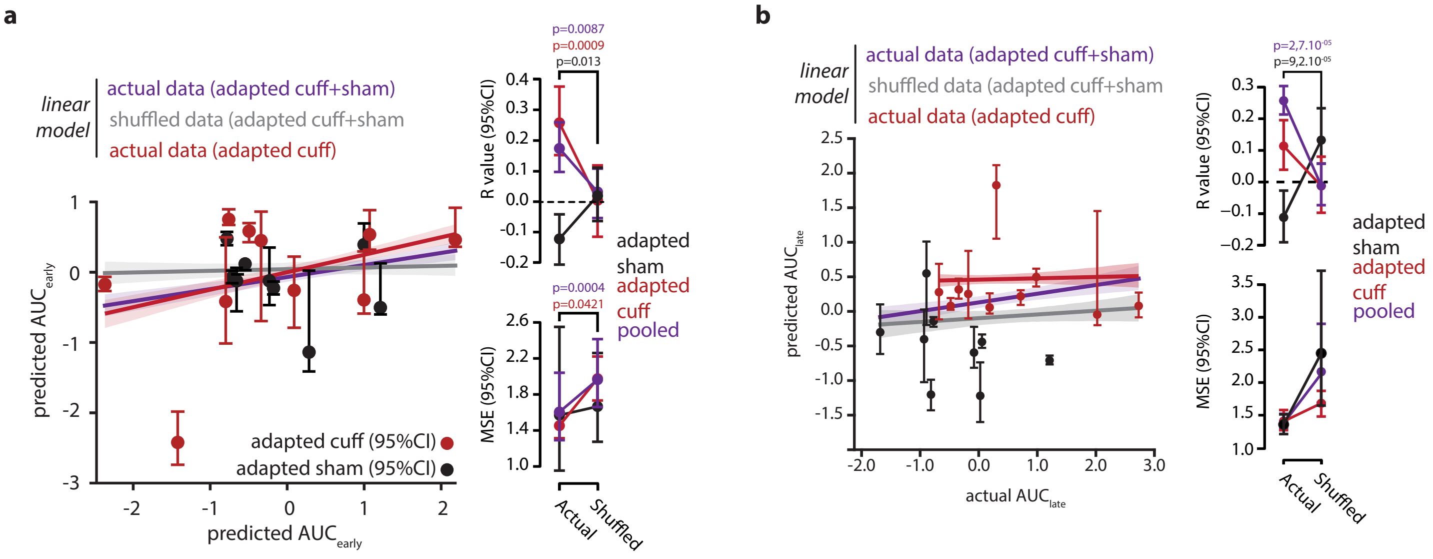

Supplementary Fig. 11 | Prediction of AUC features

Extended results of behavioral features prediction based on graph properties of synaptic maps using Generalized Linear Models (GLMs). Adapted cuff, n = 17 maps; adapted sham, n = 14 maps. When considering **(a)** early (AUC_{early}) & **(b)** late post-surgery imbalance (AUC_{late}) for prediction, GLMs predictions are worse than for previously discussed behavioral features in Figure 6. Average predictions are shown as a scatter plot on the left panel, and regression coefficients (r values) are plotted in the right panel. Linear models with actual data are shown in purple while results with shuffled data (i.e., chance level) are shown in gray. Error bars: 95% confidence intervals. Two-tailed independent t-test or two-sided MWU test between actual and shuffled R values or Mean Squared Errors. AUC_{early}: R(Pooled, actual vs shuffle) p = 0.00867; R(Adapted cuff, actual vs shuffle) p = 0.000931; R(Adapted sham actual vs shuffle) p = 0.01305; MSE(Pooled, actual vs shuffle): p = 0.000372; MSE(Adapted cuff, actual vs shuffle) p=0.0421. AUC_{late}: R(Pooled, actual vs shuffle) p = $2.696.10^{-05}$; R(Adapted sham, actual vs shuffle) r = $9.187.10^{-05}$. All p-values > 0.05 in Source Data. Source data are provided as a Source Data file.
