# Supplementary material for: Cerebellar connectivity maps embody individual adaptive behavior in mice
Source: Nat Commun. 2022 Jan 31;13:580. doi: 10.1038/s41467-022-27984-8 (PMC8803868; doi:10.1038/s41467-022-27984-8)
Supplement: Supplementary file 2 — Reporting Summary [file 41467_2022_27984_MOESM2_ESM.pdf]

nature portfolio

Corresponding author(s): Philippe Isope
Last updated by author(s): Nov 30, 2021

Reporting Summary

Nature Portfolio wishes to improve the reproducibility of the work that we publish. This form provides structure for consistency and transparency in reporting. For further information on Nature Portfolio policies, see our Editorial Policies and the Editorial Policy Checklist.

Statistics

For all statistical analyses, confirm that the following items are present in the figure legend, table legend, main text, or Methods section.

n/a	Confirmed	
☐	☒	The exact sample size (*n*) for each experimental group/condition, given as a discrete number and unit of measurement
☐	☒	A statement on whether measurements were taken from distinct samples or whether the same sample was measured repeatedly
☐	☒	The statistical test(s) used AND whether they are one- or two-sided<br>*Only common tests should be described solely by name; describe more complex techniques in the Methods section.*
☐	☒	A description of all covariates tested
☐	☒	A description of any assumptions or corrections, such as tests of normality and adjustment for multiple comparisons
☐	☒	A full description of the statistical parameters including central tendency (e.g. means) or other basic estimates (e.g. regression coefficient) AND variation (e.g. standard deviation) or associated estimates of uncertainty (e.g. confidence intervals)
☐	☒	For null hypothesis testing, the test statistic (e.g. *F*, *t*, *r*) with confidence intervals, effect sizes, degrees of freedom and *P* value noted<br>*Give P values as exact values whenever suitable.*
☒	☐	For Bayesian analysis, information on the choice of priors and Markov chain Monte Carlo settings
☒	☐	For hierarchical and complex designs, identification of the appropriate level for tests and full reporting of outcomes
☐	☒	Estimates of effect sizes (e.g. Cohen's *d*, Pearson's *r*), indicating how they were calculated

Our web collection on statistics for biologists contains articles on many of the points above.

Software and code

Policy information about availability of computer code

Data collection	Electrophysiological and behavioral data were acquired with WinWCP 4.2.2 freeware (John Dempster, SIPBS, University of Strathclyde, UK)
Data analysis	Python-based scripts that were used to analyze data and generate figures are available at https://github.com/ludo67100/CereballarMaps-GraphProperties and at https://doi.org/10.5281/zenodo.5745021<br>Python packages used are the following: Pandas 1.3, Scipy 1.6, statsmodels 0.12, Sci-kit learn 0.24, Numpy 1.19, Neo 0. 10, Orange 2.7, bctpy 0.5.2, Matplotlib 3.4, Seaborn 0.11, pingouin 0.5<br>MANOVA was performed using Real Statistics Resource Pack 6.8 for Excel version 2016

For manuscripts utilizing custom algorithms or software that are central to the research but not yet described in published literature, software must be made available to editors and reviewers. We strongly encourage code deposition in a community repository (e.g. GitHub). See the Nature Portfolio guidelines for submitting code & software for further information.

Data

Policy information about availability of data

All manuscripts must include a data availability statement. This statement should provide the following information, where applicable:

- Accession codes, unique identifiers, or web links for publicly available datasets
- A description of any restrictions on data availability
- For clinical datasets or third party data, please ensure that the statement adheres to our policy

Source data are provided with this paper. Raw data were depositied in a public repository and accessible at https://doi.org/10.5281/zenodo.5714670

Field-specific reporting

Please select the one below that is the best fit for your research. If you are not sure, read the appropriate sections before making your selection.

☒ Life sciences ☐ Behavioural & social sciences ☐ Ecological, evolutionary & environmental sciences

For a reference copy of the document with all sections, see nature.com/documents/nr-reporting-summary-flat.pdf

Life sciences study design

All studies must disclose on these points even when the disclosure is negative.

Sample size	Experiments were performed until at least 10 connectivity maps per condition could be collected. This number allowed us to use non-parametric statistical tests and was determined in agreement with our previous study Valera AM et al. Elife. 2016 Mar 16;5:e09862. doi: 10.7554/eLife.09862
Data exclusions	Recordings in which Purkinje cell leak current exceeded 1 nA were excluded as voltage-clamp measurement were not reliable
Replication	Connectivity maps recorded in control condition in adult mice replicate with success one group recorded in our previous study (Valera AM et al. Elife. 2016 Mar 16;5:e09862. doi: 10.7554/eLife.09862). All the other groups constitute new experiments and were not replicated groups
Randomization	Mice were randomly allocated to each group data as they were all from the same genetic background (Aldolase C/Venus transgenic CD1 mice)
Blinding	Experiments were not blind as cuff mice have a clear phenotype. However, blind recordings were not necessary as Purkinje cell patch-clamp and photostimulation were done similarly on acute cerebellar slices. Also in behavioral experiments, data collection was done automatically with no human scoring ruling out any experimental bias.

Reporting for specific materials, systems and methods

We require information from authors about some types of materials, experimental systems and methods used in many studies. Here, indicate whether each material, system or method listed is relevant to your study. If you are not sure if a list item applies to your research, read the appropriate section before selecting a response.

Materials & experimental systems

n/a	Involved in the study	
☒	☐	Antibodies
☒	☐	Eukaryotic cell lines
☒	☐	Palaeontology and archaeology
☐	☒	Animals and other organisms
☒	☐	Human research participants
☒	☐	Clinical data
☒	☐	Dual use research of concern

Methods

n/a	Involved in the study	
☒	☐	ChIP-seq
☒	☐	Flow cytometry
☒	☐	MRI-based neuroimaging

Animals and other organisms

Policy information about studies involving animals; ARRIVE guidelines recommended for reporting animal research

Laboratory animals	We used AldoC-venus male mice (Mus musculus) from PND9 to PND100 under CD1 background. Mice were housed with all littermates and parents from birth to weaning age (PND21). Older mice from all experimental groups were housed by 3 to 5 littermates per cage, in conditions required to fulfil their ethogram, with nesting material, as well as food and water ad libitum in a 12/12h light/dark cycle with constant hygrometry (45-50%) and temperature (21-22°C).
Wild animals	No wild animals were used
Field-collected samples	No field-collected samples were used
Ethics oversight	All experiments were conducted in accordance with the guidelines of the Ministère de l'Education Supérieure et de la Recherche and the local ethical committee, the Comité Régional En Matière d'Expérimentation Animale de Strasbourg (CREMEAS) under the agreement n° A67-2018-38 (delivered to the animal facility Chronobiotron, UMS3415, Université de Strasbourg)

Note that full information on the approval of the study protocol must also be provided in the manuscript.
